# A linkage map of *Aegilops biuncialis* reveals significant genomic rearrangements compared to bread wheat

**DOI:** 10.1002/tpg2.70009

**Published:** 2025-02-26

**Authors:** Adam Lampar, András Farkas, László Ivanizs, Kitti Szőke‐Pázsi, Eszter Gaál, Mahmoud Said, Jan Bartoš, Jaroslav Doležel, Abraham Korol, Miroslav Valárik, István Molnár

**Affiliations:** ^1^ Institute of Experimental Botany of the Czech Academy of Sciences Centre of Plant Structural and Functional Genomics Olomouc Czech Republic; ^2^ Department of Cell Biology and Genetics, Faculty of Science Palacký University Olomouc Czech Republic; ^3^ Department of Biological Resources, Agricultural Institute HUN‐REN Centre for Agricultural Research Martonvásár Hungary; ^4^ Field Crops Research Institute Agricultural Research Centre Giza Egypt; ^5^ Institute of Evolution University of Haifa Mount Carmel Israel

## Abstract

Goatgrasses with U‐ and M‐genomes are important sources of new alleles for wheat breeding to maintain yield and quality under extreme conditions. However, the introgression of beneficial traits from wild *Aegilops* species into wheat has been limited by poor knowledge of their genomes and scarcity of molecular tools. Here, we present the first linkage map of allotetraploid *Aegilops biuncialis* Vis., developed using 224 F_2_ individuals derived from a cross between MvGB382 and MvGB642 accessions. The map comprises 5663 DArTseq markers assigned to 15 linkage groups corresponding to 13 chromosomes. Chromosome 1M^b^ could not be constructed due to a lack of recombination caused by rearrangements in the MvGB382 accession. The genetic map spans 2518 cM with an average marker density of 2.79 cM. The skeleton map contains 920 segregating markers, divided between the M^b^ sub‐genome (425 markers) and the U^b^ sub‐genome (495 markers). Chromosomes of the M^b^ sub‐genome, originating from *Aegilops comosa* Sm. in Sibth. et Sm., show well‐preserved collinearity with *Triticum aestivum* L. chromosomes. In contrast, chromosomes of the U^b^ sub‐genome, originating from *Aegilops umbellulata* Zhuk., exhibit a varying degree of collinearity, with 1U^b^, 3U^b^, and 5U^b^ retaining a substantial level of collinearity with *Triticum aestivum*, while 2U^b^, 4U^b^, 6U^b^, and 7U^b^ show significant rearrangements. A quantitative trait locus affecting fertility was identified near the centromere on the long arm of chromosome 3M^b^, explaining 23.5% of the variance. The genome structure of *Aegilops biuncialis*, highlighted by the genetic map, provides insights into the speciation within the species and will support alien gene transfer into wheat.

## Core Ideas

- *Aegilops biuncialis* is a source of valuable disease resistance and stress tolerance genes for wheat breeding.
- We constructed a genetic map of *Ae. biuncialis* of 2518 cM with 5663 DArTseq markers on 15 linkage groups.
- Chromosomes of the M^b^ sub‐genome, originating from *Aegilops comosa*, retained high collinearity with *Triticum aestivum*.
- The *Aegilops umbellulata*‐derived U^b^ sub‐genome shows variable collinearity, with 2U^b^, 4U^b^, 6U^b^, and 7U^b^ most rearranged.
- The genetic map is a valuable tool to support the transfer of beneficial genes into wheat.

**Abbreviations**
CS: Chinese Spring
DArT: diversity arrays technology
FISH: fluorescence in situ hybridization
LG: linkage group
LOD: logarithm of the odds
QTL: quantitative trait locus

## 1 INTRODUCTION

Bread wheat (*Triticum aestivum* L.; 2*n* = 6*x* = 42; AABBDD) is a global staple food, securing about one‐fifth of the world's total caloric and protein intake. However, the current yield growth rate is not on track to meet food security demands due to the growing human population (Ray et al., 2013). Modern wheat's gene pool has been substantially reduced due to allopolyploid speciation, domestication, and intensive breeding. The decline in genetic variation limits the ability to breed wheat with increased yields and better tolerance to biotic and abiotic stresses. This makes the wild relatives of wheat an attractive source for enriching the wheat gene pool in introgression breeding programs (Feuillet et al., 2008).

Goatgrasses (*Aegilops*) are a rich reservoir of gene variants potentially useful for wheat improvement (Kishii, 2019; Schneider et al., 2008; P. Zhang et al., 2015). In the 11 diploid species of the genus, seven different genomes (U, M, N, C, S, D, and T) have been identified and most of them have been detected in the 12 polyploid species (Van Slageren, 1994).

Natural hybridization of diploid progenitors of U and M genomes, *Aegilops umbellulata* Zhuk. (2*n* = 2*x* = 14; UU) and *Aegilops comosa* Sm. in Sibth. et Sm. (2*n* = 2*x* = 14; MM), respectively, resulted in the formation of allotetraploid *Aegilops* species, *Aegilops biuncialis* Vis. (2*n* = 4*x* = 28; U^b^U^b^M^b^M^b^), *Aegilops geniculata* Roth. (2*n* = 4*x* = 28; M^g^M^g^U^g^U^g^), *Aegilops columnaris* Zhuk. (2*n* = 4*x* = 28; U^c^U^c^M^c^M^c^), and *Aegilops neglecta* ssp. *neglecta* Req. ex Bertol. (2*n* = 4*x* = 28; U^n^U^n^M^n^M^n^) (Kilian et al., 2011). These annual and largely autogamous species have been frequently used as gene sources in wheat introgression breeding programs (Friebe et al., 1996; Kishii, 2019; Schneider et al., 2008).

*Aegilops biuncialis* is native from the Mediterranean to West Asia, and populations can be found in Cis‐ and Transcaucasia, as well as in the southern parts of Ukraine (Kilian et al., 2011; Van Slageren, 1994). Natural habitats of *Ae. biuncialis* are characterized by annual rainfall ranging from 225 to 1250 mm (Van Slageren, 1994). Given its broad geographical distribution, high genetic diversity exists within *Ae. biuncialis* populations (Ivanizs et al., 2022, 2019). Many useful traits have been identified in *Ae. biuncialis*, yet the diversity of genebank accessions has been largely underutilized in wheat breeding (Schneider et al., 2008). Various accessions of this species are a valuable source of new genes to provide resistance to various diseases, such as barley yellow dwarf luteovirus (Makkouk et al., 1994), powdery mildew (Li et al., 2019), yellow rust (Damania & Pecetti, 1990), brown or leaf rust (Dimov et al., 1993; Kwiatek et al., 2011), and stem rust (Olivera et al., 2018), and for tolerance to abiotic stresses such as frost (Ekmekci & Terzioglu, 2002), salt (Colmer et al., 2006; Darkó et al., 2020), and drought (Dulai et al., 2014; Molnár et al., 2004). It has also been reported that *Ae. biuncialis* accessions MvGB382 and MvGB642, originating from different habitats, exhibited highly contrasting phenotypes for several agronomic traits. Compared to MvGB642, accession MvGB382 flowered 1 week earlier (Ivanizs et al., 2019), was more tolerant to drought and salt stress (Molnár et al., 2004), and improved grain quality traits such as high edible fiber (β‐glucan and water‐soluble arabinoxylan) (Rakszegi et al., 2019, 2017) and manganese and zinc content (Farkas et al., 2014). On the other hand, MvGB642 showed strong resistance to leaf rust, while MvGB382 was found to be susceptible.

To utilize the useful traits of *Ae. biuncialis* accessions MvGB642 and MvGB382 in wheat breeding programs, significant efforts have been made to transfer their U^b^ and M^b^ chromatin into wheat, resulting in bread wheat‐*Aegilops* amphiploids, disomic addition lines representing the chromosomes of 1U^b^, 2U^b^, 3U^b^, 4U^b^, 2M^b^, 3M^b^, and 7M^b^, a double disomic addition line 1U^b^ and 6U^b^, and 3M^b^(4B), 4M^b^(4D), and 5M^b^(5D) substitution and several translocation lines (Farkas et al., 2023, 2014; Molnár et al., 2009; Schneider & Molnár‐Láng, 2012; Schneider et al., 2005).

The success of *Aegilops* gene transfer to wheat is limited by several factors. An important factor is the collinearity between the homoeologous chromosomes of the donor species and wheat (Friebe et al., 1996). Evolutionarily distant species may have undergone chromosomal rearrangements, disrupting the collinearity with wheat, leading to the inhibition of meiotic chromosome pairing and wheat‐alien homoeologous recombination even in the absence of *Ph1* locus (Sears, 1977). In lines with reciprocal translocations, the loss of wheat genes may not be well compensated by the introgressed wild gene variants, leading to unbalanced genomes and reduced fitness (Devos et al., 1993; Naranjo, 2019; H. Zhang et al., 1998). Because the altered structure of the donor chromosomes suppresses recombination, the size of the introgressed chromatin segments cannot be minimized and unwanted traits/genes cannot be eliminated (Naranjo, 2019; Nasuda et al., 1998). In the case of non‐collinear chromosomes, the use of other approaches to induce random chromosome breakages, for example, ionizing irradiation (Sears, 1956) or gametocidal system such as *Aegilops cylindrica* 2C, may facilitate the creation of wheat‐alien translocations (Endo, 2007).

Chromosome‐mediated gene transfer from goatgrasses to wheat is also limited by the poor knowledge of genome structure and a lack of high‐throughput tools to screen large populations (hundreds to thousands of individuals). The segregating genetic maps facilitate the efficient utilization of genetic diversity regardless of origin (Edae et al., 2017). As reported for *Aegilops sharonensis* (Olivera et al., 2013) and *Aegilops longissima* (H. Zhang et al., 2001), a high degree of collinearity with the D genome of wheat exists for a majority of S genome chromosomes, although a 4SL/7SL translocation was detected in both species. On the other hand, wheat‐*Aegilops* comparative analysis based on F_2_ genetic maps genotyped with restriction fragment length polymorphism markers or genotyping‐by‐sequencing (GBS) markers indicated significant rearrangements of the U genome of *Aegilops umbellulata* (Edae et al., 2016, 2017; H. Zhang et al., 1998). Translocations were observed on chromosomes 4U, 5U, 6 and 7U, and chromosomes 2 and 7U contained inverted regions relative to bread wheat (Edae et al., 2017; H. Zhang et al., 1998). On the other hand, there is no information on collinearity between the M genome chromosomes and wheat, as no segregating genetic map is available for either the diploid genome progenitor *Ae. comosa* or for the allotetraploid *Ae. biuncialis*, which hinders mapping and characterization of important agronomic traits/loci.

Diversity arrays technology sequencing (DArTseq) is a genotyping‐by‐sequencing platform combining the genome complexity reduction method based on methylation‐sensitive restriction enzymes and next‐generation sequencing (Kilian et al., 2012; Sansaloni et al., 2011; Wenzl et al., 2004). This sequence‐independent, cost‐effective, and high‐throughput approach generates thousands of molecular markers of two types. Dominant Silico‐DArT markers are genomic sequences representing the presence‐absence variation of a respective fragment, whereas the co‐dominant SNP‐DArT markers indicate nucleotide polymorphism within the fragment. Both types of markers provide dense coverage of an entire genome. The DArT platform has already been used for whole‐genome profiling in *Aegilops* species, including *Aegilops tauschii* (Kumar et al., 2015), *Ae. sharonensis* (Olivera et al., 2013), and *Ae. biuncialis* (Ivanizs et al., 2022, 2019).

Motivated by the need to support genome analysis and introgression breeding, this study presents the first *Ae. biuncialis* segregating genetic map constructed using DArTseq genotyping of an F_2_ population from a cross of genebank accessions MvGB382 and MvGB642. Using the genetic map, we assessed collinearity and rearrangements of the *Ae. biuncialis* sub‐genomes compared to the reference genome of the hexaploid wheat Chinese Spring, the diploid U‐genome progenitor *Ae. umbellulata*, and the M‐genome progenitor *Ae. comosa*. Finally, by analyzing fertility, we demonstrated the suitability of the map for quantitative trait locus (QTL) mapping.

**Core Ideas**

- *Aegilops biuncialis* is a source of valuable disease resistance and stress tolerance genes for wheat breeding.
- We constructed a genetic map of *Ae. biuncialis* of 2518 cM with 5663 DArTseq markers on 15 linkage groups.
- Chromosomes of the M^b^ sub‐genome, originating from *Aegilops comosa*, retained high collinearity with *Triticum aestivum*.
- The *Aegilops umbellulata*‐derived U^b^ sub‐genome shows variable collinearity, with 2U^b^, 4U^b^, 6U^b^, and 7U^b^ most rearranged.
- The genetic map is a valuable tool to support the transfer of beneficial genes into wheat.

## 2 MATERIALS AND METHODS

### 2.1 Plant material and phenotyping

The *Ae. biuncialis* accession MvGB382 (ICAG401297) was collected in Iran (precise location not known). The accession MvGB642 (ICAG400940) was collected in Syria (Slinfah, https://www.genesys‐pgr.org/10.18730/70G4B; 1160 m above sea level; 1266 mm of mean annual precipitation). Both accessions were provided by the ICARDA genebank (Aleppo, Syria) and maintained by self‐fertilization for more than 20 years in the Department of Plant Biological Resources of HUN‐REN CAR (Martonvásár, Hungary). The mother accession MvGB382 was pollinated with the father accession MvGB642, the F_1_ hybrids were self‐pollinated and F_2_ seeds were collected in Martonvásár.

Additionally, the *Ae. umbellulata* accession AE740/03 (genome UU) and *Ae. comosa* accession MvGB1039 (genome MM), diploid progenitors of the *Ae. biuncialis* sub‐genomes, and their individual flow‐sorted chromosomes 1U–7U and 1M–7M, prepared as described by Molnár et al. (2016), were used to support anchoring of the acquired linkage groups (LGs) to chromosomes.

A total of 224 MvGB382 × MvGB642 F_2_ plants were grown in a greenhouse under conditions previously described by Rakszegi et al. (2017). Briefly, F_2_ seeds were germinated on wet filter paper at room temperature and the 8‐day‐old seedlings potted in Jiffy‐7 pellets were vernalized at 4°C for 6 weeks under 20 µmol·m^−2^·s^−1^ light intensity. After vernalization, the F_2_ seedlings were grown in 2‐L pots filled with a 3:2:1 mixture of garden soil, compost, and sand, and randomly placed in the greenhouse (Venlo Greenhouses, Kwintsheul, The Netherlands) in Martonvásár for 12 weeks. Starting from the second week after planting, each pot was fertilized with 150 mL of 0.1% complex fertilizer (Volldünger Classic) four times in total, with one application every 10 days. The initial growth condition of 11/7°C day/night temperature and 13‐h photoperiod (13‐h day/11‐h night) was gradually changed to 23/17°C day/night temperature and 16‐h photoperiod (16‐h day/8‐h night) at maturity (12 weeks). At the heading stage, the plants were isolated from each other using cellophane bags to ensure self‐fertilization. Fertility, expressed as the number of seeds per plant, was phenotyped at maturity after harvesting of each F_2_ line.

### 2.2 Genotyping and genetic map construction

Total genomic DNA was extracted from fresh leaves of 1‐week‐old plants from both parental lines, 224 F_2_ genotypes, and the *Ae. umbellulata* AE740/03 and *Ae. comosa* MvGB1039 accessions using Quick Gene‐Mini80 instrument (FujiFilm) with a QuickGene DNA Tissue Kit (FujiFilm) according to the manufacturer's instructions.

DNA samples were genotyped at Diversity Arrays Technology Pty. Ltd. (http://www.diversityarrays.com) on the Wheat DArTseq 1.0 platform. Acquired co‐dominant SNP‐DArT and dominant Silico‐DArT markers were pre‐filtered. Markers with more than 13 missing data points and segregation distortion greater than 50% of the expected allele frequency were removed. Due to the combination of dominant and co‐dominant markers, the numerical genotype codes (Silico‐DArT: 0/1; SNP‐DArT: 0/1/2) were converted to alphabetical codes (Silico‐DArT: A/C [C = B/H] or D/B [D = A/H]; SNP‐DArT: A/B/H) according to the parental genotypes using Microsoft Office Excel 2016.

To distinguish the most reliable markers during the construction of the genetic map, markers with specificity for the M genome or U genome (based on genotyping of the separate *Ae. comosa* [MM] and *Ae. umbellulata* [UU] chromosome fractions) were given a suffix “M” or “U” and corresponding chromosome groups.

The genetic map was constructed using the Multipoint Ultradense v4.2 mapping software (Ronin et al., 2017; MultiQTL Ltd., https://www.multiqtl.com/). Markers with more than 10 missing data points and *χ* ^2^  >  20 were removed. Candidate codominant “skeleton” markers representing groups of two or more co‐segregating markers were selected using the “Bound together” function (markers specific to individual chromosomes were prioritized) and clustered into LGs at the threshold of recombination fraction RF = 0.25. LGs with less than four markers were moved to the “Heap.” Each LG was manually curated until the global variation decreased below 1.2. Subsequently, the LGs were extended at their ends, and gaps were filled with additional markers from the “Heap,” if possible, using the “Extending linkage group” function with the coefficient of enlargement increased stepwise from 1.0 to 1.2. LGs with gaps larger than 30 cM were split. LGs were then tested for possible merging and those that were suitable (end‐to‐end merging, minimum recombination frequency, reasonable interval length) were merged. LGs were saturated with skeleton markers from the “Heap” that did not violate the global variation and recombination frequency and did not increase the size of the respective interval by more than 10%. After the “Heap” candidates were exhausted, the LGs were further saturated with the dominant markers. The dominant markers were inserted individually for each parental phase. Resulting LGs were exported to Microsoft Office Excel 2016 with recombination frequencies converted to centiMorgans using the Kosambi mapping function (Kosambi, 1943). The “skeleton” linkage map, which contains “skeleton” markers, that are proxies for groups of two or more co‐segregating markers, and the “global” linkage map, which contains all markers, have been exported separately.

### 2.3 Assigning linkage groups to chromosomes

To assign LGs to chromosomes and orient them, we used accessions of diploid *Ae. umbellulata* (UU) AE740/03 and *Ae. comosa* (MM) MvGB1039 and their individual flow‐sorted chromosomes. Genetic marker order was also confirmed following the wheat‐*Aegilops* homoeologous relationships previously reported for chromosomes of *Ae. umbellulata* (Edae et al., 2017; H. Zhang et al., 1998), the single‐gene map of chromosomes of *Ae. comosa* MvGB1039 and *Ae. umbellulata* AE740/03 (Said et al., 2021), and the *Ae. umbellulata* TA1851 chromosome‐scale reference assembly (Abrouk et al., 2023). LGs were assigned to *Ae. biuncialis* chromosomes 1U^b^‐7U^b^ and 2M^b^‐7M^b^, according to the recent nomenclature (Badaeva et al., 2004).

To obtain data for syntenic relationships between the LGs of the present map and the chromosomes of bread wheat, *Ae. umbellulata*, *Ae. comosa*, and *Ae. tauschii* a sequence homology search (BLASTN) (Altschul, 1997) of sequences of the mapped markers was performed on the reference genome sequence of wheat cv. Chinese Spring v2.1 (CS) (IWGSC, 2018; Zhu et al., 2021) and the assemblies of *Ae. umbellulata* TA1851 (Abrouk et al., 2023), *Ae. comosa* PI551049 (Li et al., 2024), and *Ae. tauschii* subsp. *strangulata* AL8/78 Aet v6.0 (Srikanta et al., 2024; L. Wang et al., 2021) using the blastn package of the Blast Command Line Application 2.9.0 (https://ftp.ncbi.nlm.nih.gov/). Chromosome positions of the unique best hits were used to investigate the collinearity between the LGs and wheat, *Ae. umbellulata*, *Ae. comosa*, and *Ae. tauschii* chromosomes.

Chromosome 1M^b^ was not represented by any of the LGs. Therefore, the remaining markers specific to the M genome of *Ae. comosa* and not belonging to any of the LGs were also aligned to the *Ae. comosa* chromosome 1M by BLASTN. The markers were then ordered based on their 1M chromosomal position, creating an artificial LG representing 1M^b^.

Relationships between individual chromosomes of *Ae. biuncialis*, CS, *Ae. umbellulata*, *Ae. comosa*, and *Ae. tauschii* were visualized using Circos (Krzywinski et al., 2009) within the Galaxy platform (https://usegalaxy.org/; Rasche & Hiltemann, 2020). In addition, details of the relationship between each chromosome pair can be interactively displayed in the Strudel software (available at https://ics.hutton.ac.uk/strudel/download‐strudel/; Bayer et al., 2011), for which the input data files were prepared according to the software instructions.

### 2.4 Cytogenetic verification of the 1M^b^ chromosome structure

Root tip meristem cells of *Ae. comosa* MvGB1039, *Ae. biuncialis* MvGB382, and *Ae. biuncialis* MvGB642 were synchronized using hydroxyurea, accumulated in metaphase using amiprophos‐methyl, and mildly fixed in formaldehyde (Said et al., 2019a; Vrána et al., 2016a; Vrána et al., 2016b). The synchronized root tips were used to prepare chromosome spreads for fluorescence in situ hybridization (FISH) using the drop technique described by Said et al. (2018), Said et al. (2019a), Said et al. (2019b), and Said et al. (2021).

The rye 120‐bp repeat family *pSc*119.2 (Bedbrook et al., 1980) was amplified by PCR as described by Nagaki et al. (1995). GAA microsatellites were PCR amplified from rye (cv. Daňkovské) genomic DNA with (GAA)_7_ and (CCT)_7_ primers according to Kubaláková et al. (2005), while the *Afa* family repeat (AFA) was amplified from genomic DNA of bread wheat cv. CS using AS‐A and AS‐B primers (Nagaki et al., 1995). The rice template DNA for the 5S rDNA probe was amplified by PCR according to Fukui et al. (1994).

The probes were labeled by PCR. AFA was labeled with aminoallyl‐dUTP‐CY5 (Jena Biosciences), while *pSc*119.2, GAA microsatellites, and 5S rDNA were labeled with ChromaTide Fluorescein‐12‐dUTP (Thermo Fisher Scientific). The plasmid pTa71 (45S rDNA) containing 9‐kbp fragment from *Triticum aestivum* with 18S‐5.8S‐26S rDNA and intergenic spacers (Gerlach & Bedbrook, 1979) was directly labeled by nick translation with ChromaTide Fluorescein‐12‐dUTP (Thermo Fisher Scientific).

All FISH experiments were done using combinations of two probes in each experiment, and the identity of individual chromosomes was determined based on the intensity, size, and hybridization patterns of probes as described by Said et al. (2021, 2018, 2022). Briefly, 50 ng of DNA for each tandem repeat probe in the hybridization mixture (20 µL) were added to the chromosome preparations on a microscope slide, denatured at 80°C for 3 min and hybridized overnight at 37°C. After post‐hybridization washes, the slides were mounted and counterstained with DAPI in Vectashield mounting medium (Vector Laboratories).

Chromosome preparations were examined using an Axio Imager Z.2 fluorescence microscope (Zeiss) equipped with a Cool Cube 1 camera (Metasystems) and appropriate filter sets. Signal acquisition, image processing, and chromosome measurements were performed using ISIS software (Metasystems). The final image adjustment was done in Adobe Photoshop CS5 (Adobe Systems Incorporated).

Chromosome measurements and parameter calculations were carried out as described by Said et al. (2021, 2018). Briefly, the 10 best images of mitotic metaphase spreads obtained at 100× magnification were selected and used. Chromosomes were measured in micrometer, and the average of 10 measurements was used to calculate the Centromeric index and the Arm ratio (L/S) for each chromosome. Standard deviations and confidence intervals with a significance level of 0.05 were calculated using Microsoft Office Excel 2016 functions.

### 2.5 Quantitative trait loci analysis

A QTL for fertility was mapped by interval mapping using the MultiQTL v2.6 software package (Korol et al., 2009; MultiQTL Ltd.) employing the skeleton linkage map containing only the most informative markers.

The significance of the detected QTL effect was tested by permutation test (10,000 iterations), and the significant model was analyzed by bootstrap analysis (10,000 iterations) to estimate the standard deviation of the QTL effect and its chromosomal position. The effect of the QTL was estimated as the percentage of explained variance of the trait relative to its phenotypic variation.

## 3 RESULTS

### 3.1 Genetic map

The DArTseq analysis resulted in 53,394 markers (6273 codominant SNP‐DArT and 47,121 dominant Silico‐DArT markers) obtained for 224 F_2_ individuals and their parental lines (*Ae. biuncialis* MvGB382 and MvGB642). The genotyping also included diploid progenitors of U^b^ and M^b^ *Ae. biuncialis* sub‐genomes (*Ae. comosa* MvGB1039 and *Ae. umbellulata* AE740/03), DNAs of their individual flow‐sorted chromosomes 1M–7M and 1U–7U, and hexaploid wheat cv. CS.

Marker data of the 224 F_2_ individuals were processed using the Multipoint Ultradense v4.2 mapping software and 25,785 markers passed the quality filtering. After the first clustering, 931 marker groups were created with a total of 6411 markers. The segregating codominant SNP markers were used to construct a skeleton map, resulting in 15 LGs with chromosomes 5U^b^ and 6U^b^ each represented by two LGs (Table 1, Data S1 and S2). The final map contains 3336 (codominant) SNP‐DArT and 2327 (dominant) Silico‐DArT markers.

**TABLE 1:** Characterization of *Aegilops biuncialis* MvGB382 × *Ae. biuncialis* MvGB642 genetic map.

Chr.	Length (cM)	Total no. of markers	No. of skeleton markers	Skeleton marker density (cM)
1M^b^ ^1^	–	(43)	–	–
2M^b^	172.44	801	83	2.01
3M^b^	209.13	549	82	2.52
4M^b^	141.03	390	58	2.43
5M^b^	225.49	677	86	2.62
6M^b^	149.61	483	66	2.27
7M^b^	125.39	428	50	2.51
1U^b^	149.78	328	62	2.42
2U^b^	226.82	333	66	3.44
3U^b^	145.59	326	59	2.47
4U^b^	158.13	306	43	3.68
5U^b^‐1^2^	119.76	246	57	2.10
5U^b^‐2^2^	138.59	147	48	2.89
6U^b^‐1^3^	164.62	200	50	3.29
6U^b^‐2^3^	191.40	245	53	3.61
7U^b^	200.30	204	57	3.51
M^b^ genome^4^	1023.09	3328	425	2.39
U^b^ genome	1494.99	2335	495	3.05
Total^4^	2518.08	5663	920	2.79

*Note*: Chromosomes 5U^b^ and 6U^b^ are each represented by two linkage groups.

^1^ Chromosome 1M^b^ was created artificially. The markers were ordered based on the synteny with the 1D chromosome of CS v2.1 (IWGSC, 2018; Zhu et al., 2021).

^2^ The 5U^b^‐1 linkage group (LG) corresponds to chromosome 5US and the proximal part of chromosome 5UL, while the 5U^b^‐2 LG corresponds to the distal part of chromosome 5UL.

^3^ The 6U^b^‐1 LG corresponds to chromosome 6US and the 6U^b^‐2 LG corresponds chromosome 6UL.

^4^ Excluding the artificial 1M^b^ LG.

No part of the 1M^b^ chromosome was represented by any of the LGs. As the previous single‐gene FISH map of *Ae. comosa* showed 1M–1D collinearity (Said et al., 2021), we created an artificial LG for chromosome 1M^b^ using markers mapped on the chromosome 1M of *Ae. comosa* PI551049 (Li et al., 2024) and unassigned to any other LG. These markers were ordered based on their position on chromosome 1M (Data S1).

The total length of the *Ae. biuncialis* MvGB382 × *Ae. biuncialis* MvGB642 genetic map is 2518.08 cM (Table 1) and comprises 920 segregating “skeleton” markers and 4743 co‐segregating “attached” markers (excluding chromosome 1M^b^). The average distance between two skeleton markers is 2.39 cM for the M^b^ genome and 3.05 cM for the U^b^ genome. The largest gap is 21.26 cM long. The 7M^b^ chromosome was found to be the shortest (125.39 cM), while 6U^b^, composed of two LGs, is the longest (356.02 cM). The total length of the M^b^ genome (1023.09 cM) was shorter compared to the U^b^ genome (1494.99 cM), and the number of skeleton markers was also lower (425 and 495 markers for the M^b^ and U^b^ sub‐genomes, respectively), most likely due to the absence of the 1M^b^ LG.

### 3.2 Chromosome 1M^b^ structure analysis

**FIGURE 1:**
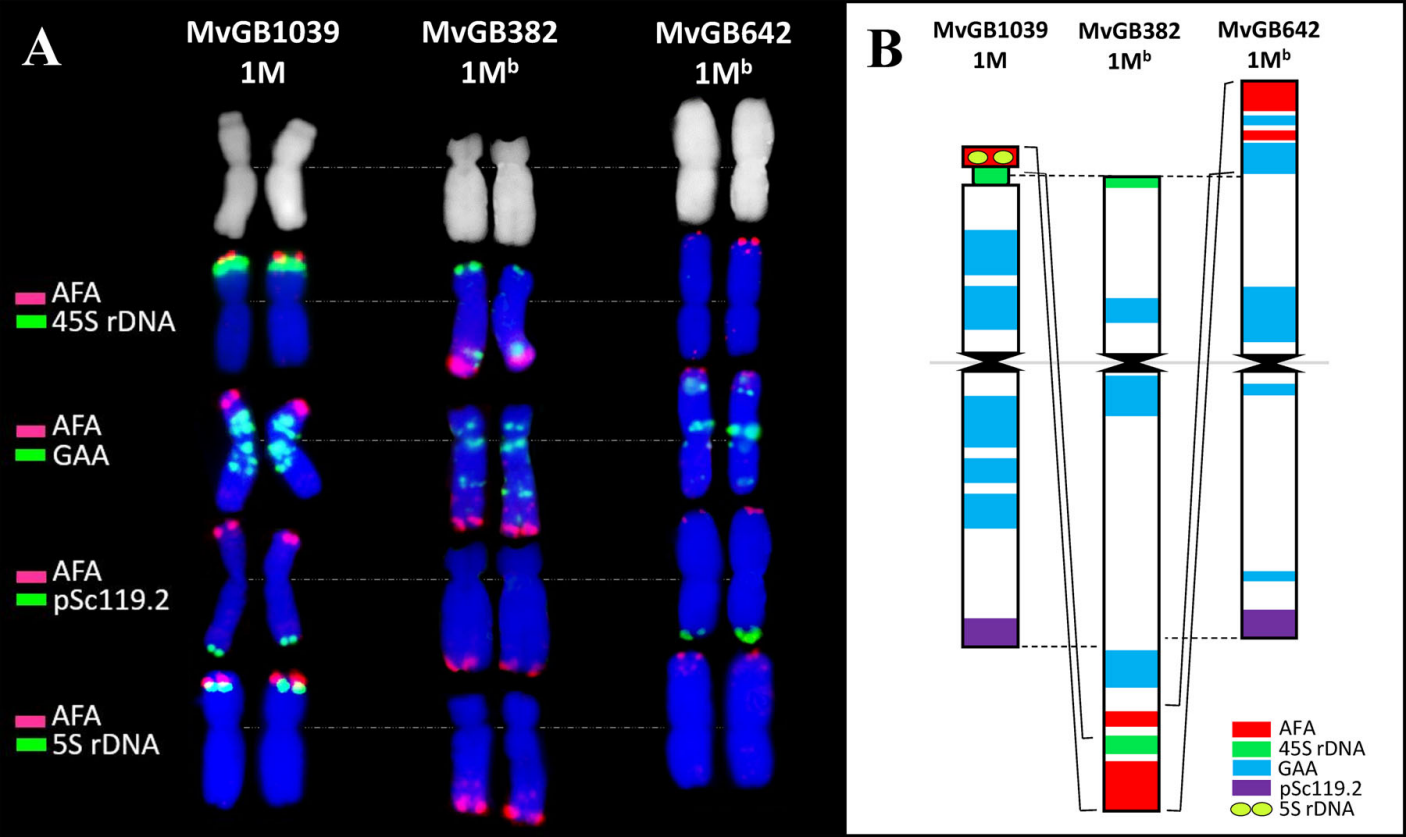
Schematic representation of structural rearrangements of chromosome 1M of *Aegilops comosa* MvGB1039 and 1M^b^ of *Aegilops biuncialis* MvGB382 and *Ae. biuncialis* MvGB642. (A) Fluorescence in situ hybridization (FISH) on mitotic metaphase chromosomes 1M and 1M^b^ from the abovementioned accessions using probes for *Afa* family repeat (red) and probes for 45S rDNA, GAA microsatellites, pSc119.2 repeat, and 5S rDNA (green). Chromosomes were counterstained with DAPI (blue). (B) Idiogram showing a possible translocation of the distal part of the short arm of chromosome 1M^b^ of MvGB382 to the long arm of the same chromosome. The distal fragment of the short arm of 1M^b^ of MvGB642 underwent further evolutionary reorganizations, losing the secondary constriction and the 45S rDNA sequence repeats.

To explain why the 1M^b^ LG could not be constructed, we compared the structure of the 1M chromosomes from both *Ae. biuncialis* parental lines and *Ae. comosa* MvGB1039 using molecular cytogenetic approaches (Figure 1A).

The lengths of the *Ae. biuncialis* 1M^b^ chromosomes were comparable and slightly longer (14% in average) compared to the *Ae. comosa* 1M chromosome (Table 2). However, we found that the positions of the centromeres were different. The 1M^b^ chromosome of MvGB382 was found to be submetacentric, while the 1M chromosomes of MvGB642 and MvGB1039 were metacentric.

**TABLE 2:** Chromosome 1M parameters in *Aegilops comosa* MvGB1039, *Ae. biuncialis* MvGB382 and *Ae. biuncialis* MvGB642.

Chromosome	MvGB1039 (1M)	MvGB382 (1M^b^)	MvGB642 (1M^b^)
Chromosome length (µm) ± SD	6.53 ± 0.07	7.61 ± 0.38	7.22 ± 0.12
Short arm (µm) (S) ± SD	2.91 ± 0.06	1.78 ± 0.08	3.88 ± 0.07
Long arm (µm) (L) ± SD	3.62 ± 0.05	5.83 ± 0.32	3.34 ± 0.08
Arm ratio (L/S)	1.24	3.28	0.86
Centromeric index (S/1M) × 100	44.56	23.39	53.74

The structure of the MvGB1039, MvGB382, and MvGB642 1M chromosomes was further examined by FISH (Figure 1A). Signals from all probes showed different intensity, size, and hybridization patterns on all 1M tested chromosomes. The structure of the chromosomes was examined using five FISH probes. The AFA probe signal was located at the distal end of the long arm of the MvGB382 chromosome 1M^b^ and at the distal end of the short arm of chromosome 1M of MvGB1039 and chromosome 1M^b^ of MvGB642. The 45S rDNA probe signal was detected at the distal end of the short arm of the MvGB382 and MvGB1039 1M chromosomes and at the distal end of the long arm of the MvGB382 1M^b^ chromosome. No 45S rDNA probe was detected on the MvGB642 1M^b^ chromosome. The signals of the probes for GAA microsatellites were detected in the pericentromeric regions of both arms of all tested chromosomes. The MvGB382 and MvGB642 1M^b^ chromosomes also have signals at the distal ends of the long arms. The probe was also detected at the distal end of the short arm of the MvGB642 1M^b^ chromosome contrary to the MvGB382 1M^b^ chromosome. The pSc119.2 repeat probe signal was detected at the distal ends of the long arms of the MvGB1039 and MvGB642 1M chromosomes. The signal of the 5S rDNA cluster was detected at the distal end of the short arm of the *Ae. comosa* MvGB1039 chromosome 1M and overlaps with the position of the 45S rDNA probe. However, unlike the 45S rDNA probe, the 5S rDNA probe was not detected on the MvGB382 1M^b^ chromosome.

### 3.3 Comparison of the *Aegilops biuncialis* chromosomes with bread wheat and *Aegilops tauschii* reference genomes

**FIGURE 2:**
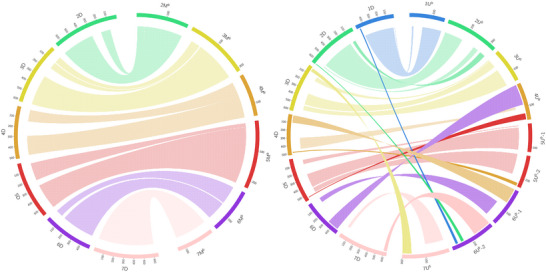
Syntenic relationships between the *Aegilops biuncialis* M^b^ and U^b^ sub‐genomes and the CS D sub‐genome. Dotted ribbons (the shorter ribbons) connect collinear segments between the same chromosomal groups. Solid ribbons (the longer ribbons) connect collinear segments between different chromosomal groups. Ribbons with horizontal lines (the shorter ribbons) or vertical lines (the longer ribbons) connect inverted segments between the same or different chromosomal groups, respectively. Chromosome 1M^b^ is not shown because the 1M^b^ LG could not be constructed. The CS chromosomes are in Mb, while the M^b^ and U^b^ chromosomes are in cM.

The DArTseq markers from the linkage map were mapped to the reference sequence of CS (IWGSC, 2018; Zhu et al., 2021). Out of 5663 markers, 2738 markers were mapped to the reference, which was used to assess the syntenic relationships between *Ae. biuncialis* and hexaploid wheat. The highest number of markers (1003) was mapped to the A genome of CS compared to 982 and 753 markers mapped to the D and B genomes, respectively. The 1U^b^, 2M^b^, 3M^b^, 3U^b^, 4M^b^, 5M^b^, 5U^b^‐1, 5U^b^‐2, 6M^b^, and 7M^b^ LGs have well‐preserved collinearity with CS chromosomal groups 1, 2, 3, 4, 5, 6, and 7, respectively. Synteny with CS D sub‐genome is shown in Figure 2 and synteny with A and B sub‐genomes in Data S3 (detailed chromosome relationships are available in Data S4 and S5). The remaining LGs show a certain degree of rearrangement in comparison to CS.

The distal end of the long arm of chromosome 2U^b^ has a translocated segment (191–218 cM) syntenic with the short arm of chromosomes 2A, 2B, and 2D (corresponding to 41 Mb on chromosome 2D). The 1–70 cM part of chromosome 4M^b^ is syntenic with almost the entire chromosome 4A (except the terminal end of the 4AL arm) in an inverted orientation, while the collinearity between chromosome 4M^b^ and chromosomes 4B and 4D is well preserved. The 4U^b^ LG is syntenic with several CS chromosomes (Figure 2, Data S4 and S5). The 0–93 cM region maps to the long arms of group 6 chromosomes in an inverted orientation. The adjacent part (93–135 cM) corresponds to the homologous parts of 4AS, 4BL, and 4DL. The remaining part of the 4U^b^L corresponds to the distal part of chromosomes 5AL, 5BL, and 5DL. The 5M^b^, 5U^b^‐1, and 5U^b^‐2 LGs show collinearity with chromosomes 5A, 5B, and 5D. However, the distal end of the long arm of chromosome 5M^b^ is syntenic with the distal end of the long arm of chromosome 4A (in an inverted orientation) and the distal end of the long arm of chromosome 5U^b^ is syntenic with the distal ends of the long arms of chromosomes 4B and 4D. Markers on the short arm of chromosome 6U^b^ (the 6U^b^‐1 LG) mapped to CS chromosomal groups 4 and 6. The 0–70 cM part is composed of markers syntenic with the long arm of chromosome 4A (90 Mb) and short arms of chromosomes 4B and 4D in an inverted orientation (100 Mb). The 81–163 cM part contains markers syntenic with the short arms of chromosomes 6A, 6B, and 6D (45–140 Mb). The long arm of chromosome 6U^b^ (the 6U^b^‐2 LG) contains markers collinear with CS chromosomal groups 7, 2, and 1. The 1–82 cM part is collinear with the distal ends of the long arms of group 7 chromosomes (43–74 Mb). The 108–146 cM part is collinear with the distal ends of the long arms of group 2 chromosomes (23 Mb), and the 162–187 cM part is collinear with the distal parts of the long arms of group 1 chromosomes (6–35 Mb). The 7U^b^ LG covers a part of the long arm of chromosome 7U^b^. The short arm and the proximal part of the long arm were not constructed. The 1–152 cM part is syntenic with the short arms of chromosomes 7A, 7B, and 7D (10–80 Mb) in an inverted orientation, while the 160–200 cM part is syntenic with the short arms of chromosomes 3A, 3B, and 3D (42–66 Mb) in an inverted orientation. A part (10–60 cM) of this LG is also syntenic with the distal part of the long arm of chromosome 4A (46 Mb) in an inverted orientation.

The DArTseq markers were also mapped to the reference sequence of *Ae. tauschii* (Aet v6.0, Srikanta et al., 2024) (graph available in Data S3 and detailed relationships in Data S1, S4, and S6). A total of 1277 out of 5663 markers were mapped to the reference, which is a higher number compared to the number of markers mapped to the CS D sub‐genome (982). The syntenic relationships are the same as those between *Ae. biuncialis* and the CS D sub‐genome, with differences in resolution being caused by a different number of mapped markers.

### 3.4 Comparison of *Aegilops biuncialis* chromosomes with the *Aegilops umbellulata* and *Aegilops comosa* genome assemblies

**FIGURE 3:**
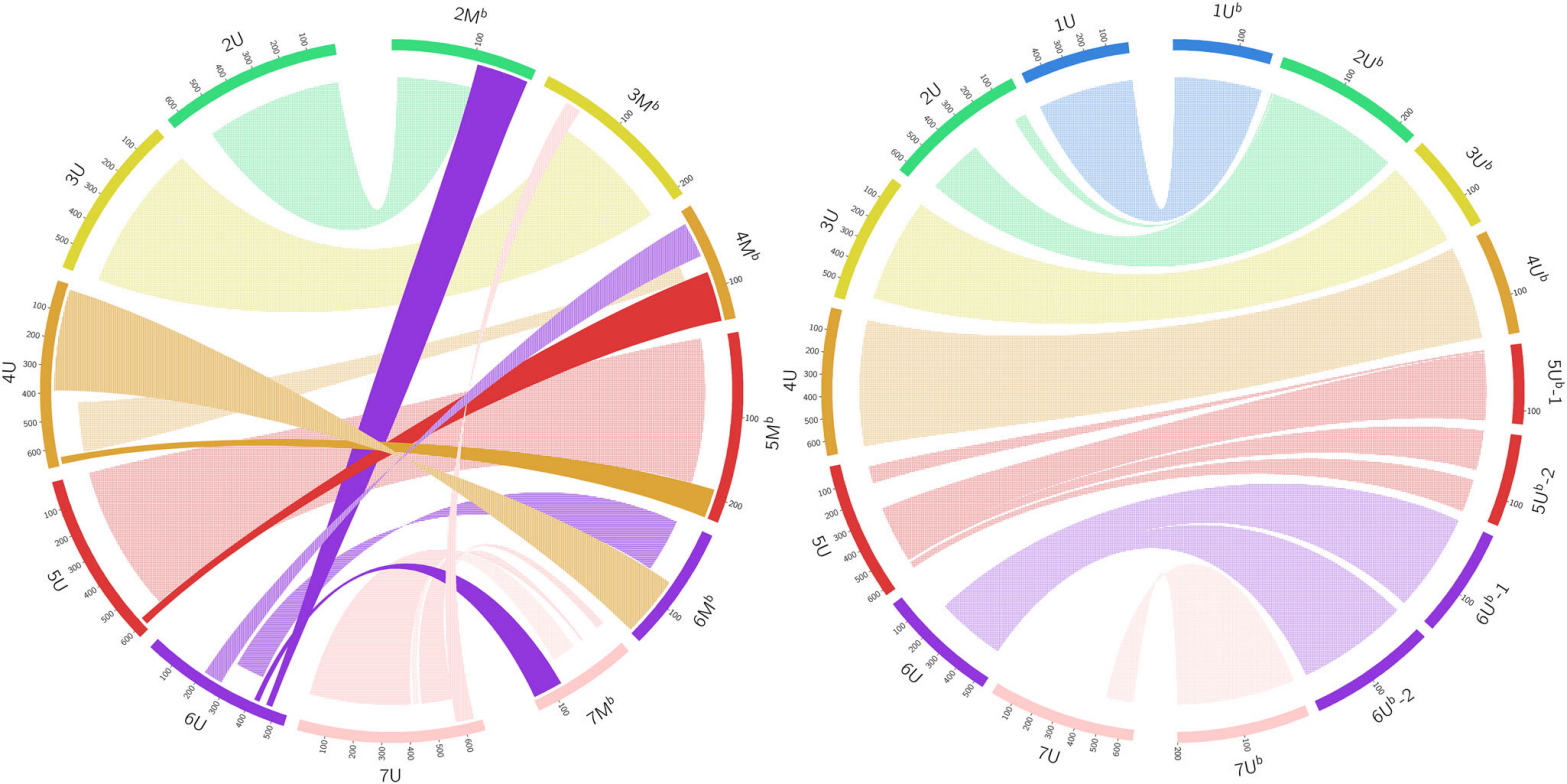
Syntenic relationships between the *Aegilops biuncialis* M^b^ and U^b^ sub‐genomes and *Aegilops umbellulata* TA1851. Dotted ribbons (the shorter ribbons) connect collinear segments between the same chromosomal groups. Solid ribbons (the longer ribbons) connect collinear segments between different chromosomal groups. Ribbons with horizontal lines (the shorter ribbons) or vertical lines (the longer ribbons) connect *inverted* segments between the same or different chromosomal groups, respectively. Chromosome 1M^b^ is not shown because the 1M^b^ LG could not be constructed. The *Ae. umbellulata* chromosomes are in Mb, while the M^b^ and U^b^ chromosomes are in cM.

To assess the synteny with *Ae. umbellulata*, 2728 markers were mapped to its reference sequence TA1851 (Abrouk et al., 2023). The *Ae. biuncialis* U^b^ sub‐genome chromosomes are all collinear with the respective chromosomes of *Ae. umbellulata*, while the M^b^ sub‐genome chromosomes show rearrangements (Figure 3, Data S1, S4, and S7).

The U^b^ sub‐genome LGs cover most of the U genome of TA1851, except for the 7U^b^ LG, which is collinear with only a 510–644 Mb region of the long arm of 7U.

The short arm and the proximal part of the long arm of chromosome 2M^b^ are collinear with 2U, but the distal end of the long arm is collinear with the distal end of the long arm of chromosome 6U. The distal part of the short arm of chromosome 3M^b^ (4–53 cM) is syntenic with the distal end of the long arm of chromosome 7U in an inverted orientation. The remaining part of chromosome 3M^b^ is collinear with chromosome 3U. The short arm of chromosome 4M^b^ is syntenic with the short arm of chromosome 6U in an inverted orientation. The proximal part of the long arm of chromosome 4M^b^ (59–80 cM) is collinear with the long arm of chromosome 4U and the distal part of the long arm of chromosome 4M^b^ is collinear with the distal end of the long arm of chromosome 5U. Chromosome 5M^b^ is collinear with chromosome 5U except for the distal end of the long arm, which is collinear with the distal end of the long arm of chromosome 4U. The short arm of chromosome 6M^b^ is syntenic with the proximal part of the long arm of chromosome 6U in an inverted orientation, while the long arm of chromosome 6M^b^ is syntenic with the long arm of chromosome 4U in an inverted orientation. The distal part of the short arm of chromosome 7M^b^ is syntenic with the distal part of the long arm of chromosome 7U. The proximal part of the long arm of chromosome 7M^b^ is syntenic to a pericentromeric region on the long arm of chromosome 7U, suggesting a translocation within this arm. The distal end of chromosome 7M^b^ is collinear with a part of the long arm of chromosome 6U (414–432 Mb).

**FIGURE 4:**
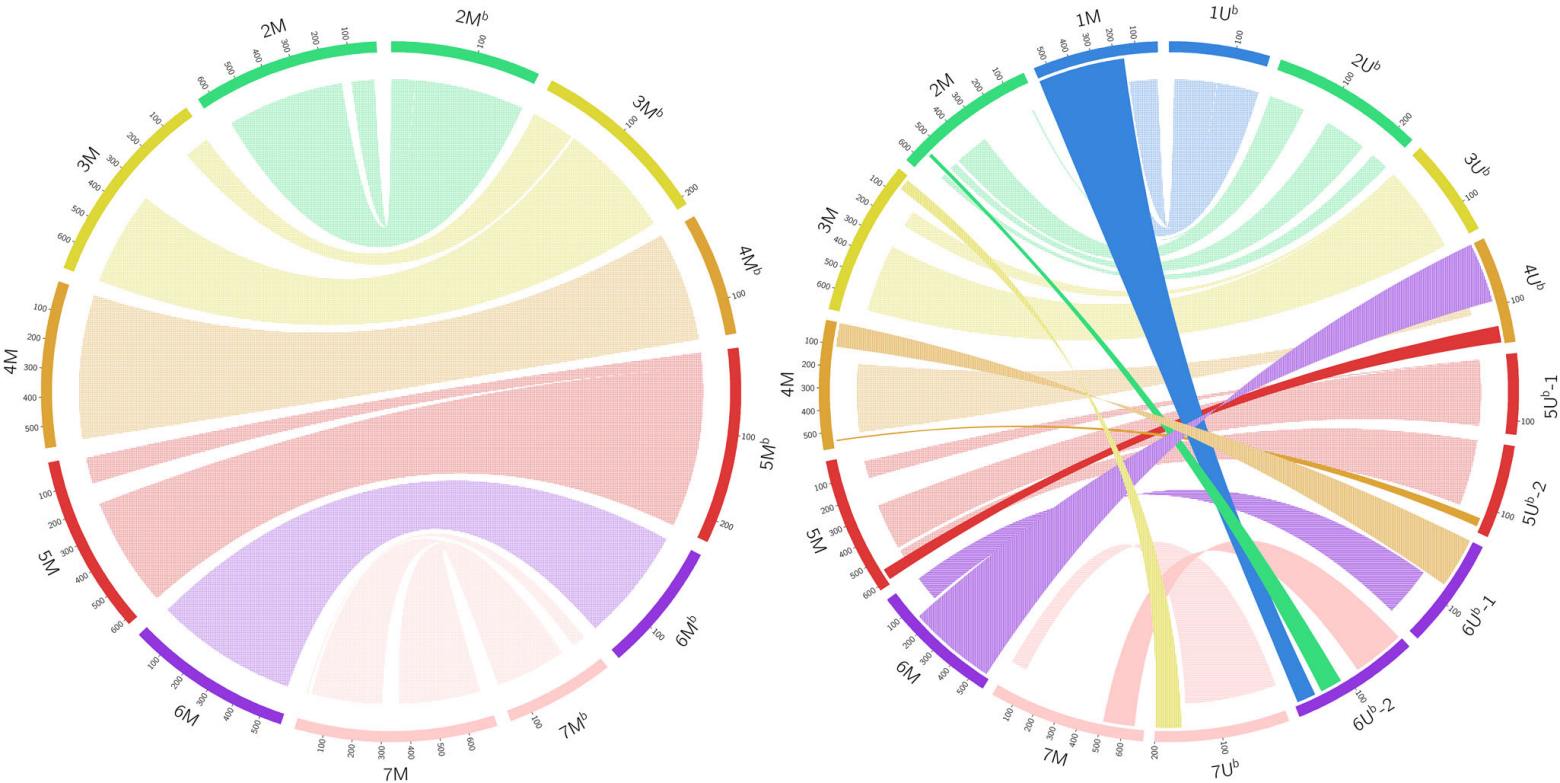
Syntenic relationships between the *Aegilops biuncialis* M^b^ and U^b^ sub‐genomes and *Aegilops comosa* PI551049. Dotted ribbons (the shorter ribbons) connect collinear segments between the same chromosomal groups. Solid ribbons (the longer ribbons) connect collinear segments between different chromosomal groups. Ribbons with horizontal lines (the shorter ribbons) or vertical lines (the longer ribbons) connect inverted segments between the same or different chromosomal groups, respectively. Chromosome 1M^b^ is not shown because the 1M^b^ LG could not be constructed. The *Ae. comosa* chromosomes are in Mb, while the M^b^ and U^b^ chromosomes are in cM.

Similar to *Ae. umbellulata*, 2913 markers were mapped to the reference sequence of *Ae. comosa* PI551049 (Li et al., 2024). The *Ae. biuncialis* M^b^ sub‐genome is collinear with the genome of *Ae. comosa*, while the U^b^ sub‐genome shows rearrangements (Figure 4, Data S1, S4, and S8).

The M^b^ sub‐genome LGs cover all *Ae. comosa* chromosomes, excluding 1M because the 1M^b^ LG could not be constructed.

Chromosomes 1U^b^, 3U^b^, and 5U^b^ are collinear with 1M, 3M, and 5M, respectively (5U^b^‐1 LG with the 37–494 Mb region and 5U^b^‐2 LG with the 513–579 Mb region of chromosome 5M). Chromosome 2U^b^ is collinear with chromosome 2M, but there are only a few markers that map to the short arm of chromosome 2M, and there are no markers from 40 to 310 Mb, with the centromeric region located at about 190 Mb (Li et al., 2024). Similarly to 4M^b^, the short arm of 4U^b^ is syntenic with chromosome 6M in an inverted orientation. However, it covers not only the proximal region of the short arm of chromosome 6M, but also the long arm. A part of the long arm of chromosome 4U^b^ (91–105 cM) is collinear with the long arm of chromosome 4M and the distal part of the long arm of chromosome 4U^b^ is collinear with the distal end of the long arm of chromosome 5M. The 6U^b^‐1 LG markers were mapped to chromosomes 4M and 6M. The 0–77 cM part syntenic with the short arm of chromosome 4M (1–110 Mb) in an inverted orientation. The 81–162 cM part contains markers syntenic with the short arm of chromosome 6M (9–134 Mb) in an inverted orientation. The long arm of chromosome 6U^b^ (the 6U^b^‐2 LG) contains markers collinear with chromosomes 7, 2, and 1. The 2–82 cM part is collinear with the distal end of the long arm of chromosome 7M (654–693 Mb). The 112–146 cM part is collinear with a part of the long arm of chromosome 2M (549–564 Mb), and the 157–187 cM part is collinear with the distal part of the long arm of chromosome 1M (510–529 Mb). The 1–152 cM part of the 7U^b^ LG is syntenic with the distal part of the short arm of chromosome 7M in an inverted orientation (2–83 Mb) and the 160–200 cM part is syntenic with the distal part of the short arm of chromosome 3M in an inverted orientation (17–66 Mb).

Comparing the genome assemblies of *Ae. umbellulata* and *Ae. comosa* (Abrouk et al., 2023; Li et al., 2024) with the obtained LGs for the U^b^ and M^b^ sub‐genomes of *Ae. biuncialis*, the U^b^ LGs represent 68% of the U genome of *Ae. umbellulata* and the M^b^ LGs represent 74% of the M genome of *Ae. comosa* (excluding the artificially constructed chromosome 1M^b^).

### 3.5 QTL mapping

**FIGURE 5:**
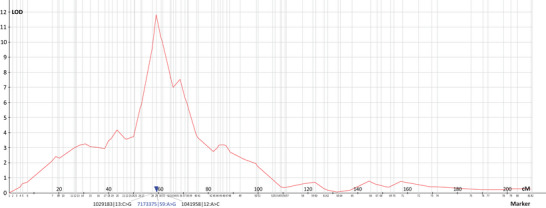
QTL *QFert.ieb‐3M^b^* associated with fertility mapped in the *Aegilops biuncialis* MvGB382 × *Ae. biuncialis* MvGB642 F_2_ population. The quantitative trait locus (QTL) for fertility, *QFert.ieb‐3M^b^*, was mapped on chromosome 3M^b^ with a logarithm of the odds (LOD) score of 11.8 (Table 3), with a peak association at marker *7173375|59:A >* *G*, located within the region flanked by markers *1029183|13:C >* *G* and *1041958|12:A >* *C*. The numbers on the *x*‐axis correspond to the 3M^b^ linkage group marker order provided in Supporting Information Data 1.

A QTL for fertility, phenotyped as the number of seeds per plant and designated as *QFert.ieb‐3M^b^*, was detected on chromosome 3M^b^. The QTL peak was located near marker *7173375|59:A >* *G*, corresponding to a position of 325 Mb on chromosome 3D of CS (Zhu et al., 2021), within a 3.4 cM region flanked by markers *1029183|13:C >* *G* and *1041958|12:A >* *C* (Figure 5). The QTL had a logarithm of the odds (LOD) score of 11.8 and explained 23.5% of the variance for this trait (Table 3). In the studied population, the number of seeds per plant ranged from 0 to 103 seeds (parental lines MvGB642 and MvGB382 had 99 and 63 seeds, respectively). The positive effect of the QTL was contributed by the MvGB642 parent (Table 3).

**TABLE 3:** Quantitative trait locus mapped for fertility in *Aegilops biuncialis* MvGB382 × *Ae. biuncialis* MvGB642 F_2_ population.

Trait	QTL	Chr.	QTL peak location ± SD (cM)	QTL location (95% confidence interval) (cM)	LOD ± SD	LOD threshold (Confidence level 99.9%)	PEV ± SD (%)	Substitution effect (d)^1^ ± SD
Fertility	*QFert.ieb‐3M^b^*	3M^b^	59.7 ± 4.1	51.6‐67.7	11.8 ± 2.7	4.4	23.5 ± 4.7	1.1 ± 5.8

Abbreviations: LOD, logarithm of the odds; PEV, percentage of explained variance; QTL, quantitative trait locus.

^1^ Substitution effect (d): direction + positive effect of the MvGB642 allele, direction ‐ positive effect of the MvGB382 allele.

## 4 DISCUSSION

Tetraploid goatgrass *Ae. biuncialis* is an attractive source of new genes and alleles to enrich the bread wheat gene pool. Identifying positions of the loci containing agronomically important genes is a prerequisite for their efficient use in breeding. Moreover, the *Ae. biuncialis* genetic map could facilitate the detection and characterization of U‐ and M‐genome chromosomal segments in the genetic background of wheat and high‐throughput selection of wheat‐*Ae. biuncialis* introgression lines as recently demonstrated in *Ae. biuncialis* (Gaál et al., 2024), *Aegilops mutica*, and *Aegilops speltoides* (King et al., 2016, 2017). Using an axiom 35K SNP array optimized for detecting polymorphisms between wheat and its wild relatives, King et al. (2016) developed a 729 cM map of *Ae. mutica* containing 613 SNP markers and a 595 cM map of *Ae. speltoides* with 544 SNPs (King et al., 2017) and detected 218 wheat‐*Ae. mutica* and 294 wheat‐*Ae. speltoides* introgressions. Gaál et al. (2024) employed DArTseq to screen 79 individuals to detect introgressed *Ae. biuncialis* MvGB382 and MvGB642 chromatin in the genetic background of parental wheat accession Mv9kr1 using 8226 and 4266 markers specific for the U^b^ and M^b^ sub‐genomes, respectively, and effectively identified disomic addition lines representing chromosomes 4M^b^ and 5M^b^.

Here, we present the first genetic map of *Ae. biuncialis* based on a mapping population of 224 F_2_ individuals. The map consists of 920 segregating “skeleton” markers, representing groups of two or more co‐segregating markers, and 4743 co‐segregating “attached” markers. The total length is 2518 cM (Table 1, Data S1), which is comparable with the map of tetraploid *Triticum durum* (2317 cM, Peleg et al., 2008).

Fifteen LGs identified in this study correspond to thirteen *Ae. biuncialis* chromosomes (excluding the 1M^b^ chromosome), anchored based on marker collinearity with the CS reference genome (IWGSC, 2018; Zhu et al., 2021) and the recent *Ae. umbellulata* and *Ae. comosa* reference genome sequence assemblies (Abrouk et al., 2023; Li et al., 2024). The constructed LGs for U^b^ and M^b^ sub‐genomes represent 68% of the U genome of *Ae. umbellulata* and 74% of the M genome of *Ae. comosa* (excluding chromosome 1M^b^), respectively. This comparatively lowered coverage is due to the absence of LGs corresponding to the pericentromeric region of 2U, parts of the short arms of 5 and 6U, the short arm and a part of the long arm of 7U, whole chromosome 1M, and a part of the short arm of chromosome 3M. These gaps are most likely caused by the absence of markers or, as in the case of chromosome 1M^b^, by limited recombination in these regions.

The pairs of the 5U^b^ and 6U^b^ LGs form complete chromosomes divided by significant gaps (>20 cM). The gap on chromosome 6U^b^ is in the pericentromeric region and could be a result of a combination of a low level of polymorphism within the mapping population and the presence of large stretches of heterochromatin that are not covered by DArTseq markers due to the generation of genome representation using a methylation‐sensitive DNA restriction endonuclease (Wenzl et al., 2004). The reason for the presence of the gap on chromosome 5U^b^, located in the distal region of the long arm and analogous to the region of about 497–517 Mb on chromosome 5U of the TA1851 *Ae. umbellulata* and 5M of the PI551049 *Ae. comosa* assemblies, is unclear.

A similar situation was also observed for the 1U chromosome in the map of *Ae. umbellulata* (Edae et al., 2016), the ancestor of the *Ae. biuncialis* U^b^ genome, suggesting a lower level of diversity within the *Ae. umbellulata* species compared to cultivated wheat. In cultivated wheat, such large gaps have typically been the result of insufficient marker density in these regions. With the advent of high‐throughput genotyping platforms, such as the 90k SNP chip (Balcárková et al., 2017; S. Wang et al., 2014) or DArTseq (Cruz et al., 2013; Korchanová et al., 2022) and GBS (Elshire et al., 2011) platforms, large gaps within chromosomes are becoming rare.

The number of the U^b^ and M^b^ skeleton markers is similar, but the total length of the M^b^ LGs is only 68% of the U^b^ LGs. Edae et al. (2016) constructed an *Ae. umbellulata* SNP map with a total length of 932 cM, which is 563 cM less than the length of our U^b^ genome map. This discrepancy occurred mainly due to the presence of two LGs for each of the 5U^b^ and 6U^b^ chromosomes. The length of the LGs and gaps suggest a high frequency of recombination, which is in strong contrast to observations on chromosome 1M^b^.

The 1M^b^ chromosome was not represented by any LG assembled based on recombination frequency. Cytogenetic analysis (FISH) of the 1M^b^ chromosome structure of both MvGB382 and MvGB642 accessions and comparison with the *Ae. comosa* MvGB1039 1M chromosome revealed significant chromatin reconstructions (Figure 1A). While the length of the MvGB382 and MvGB642 1M^b^ chromosomes was similar (7.6 and 7.2 µm, respectively), they were 14% longer compared to the 1M chromosome (Table 2). Nevertheless, the MvGB382 1M^b^ chromosome is submetacentric, while the MvGB642 1M^b^ chromosome is metacentric (Figure 1A). The difference in the position of the centromere, together with the positions of the AFA, 45S rDNA, and pSc119.2 probes, indicates that the MvGB382 1M^b^ chromosome is rearranged compared to the 1M chromosomes of MvGB1039 and MvGB642. This rearrangement could be a result of a translocation involving a breakage at the subtelomeric position of the MvGB382 1M^b^S chromosomal arm, followed by an inversion and reattachment to the long arm of the same chromosome (Figure 1B). The breakpoint occurred within the 45S rDNA region, as evidenced by the remaining 45S rDNA signals at the telomeric short arm and the subtelomeric long arm of this chromosome.

Such significant structural differences are known to interfere with chromosome pairing and inhibit meiotic recombination in wheat (Majka et al., 2023), causing gamete abortion and acting as a barrier to gametes with unrecombined chromosomes. Correct recombination is therefore essential for a successful meiotic cycle (reviewed in Roeder, 1997). We hypothesize that meiotic cycle in the MvGB642 × MvGB382 F_1–2_ progenies could have been completed in three different ways: (i) completion even without the recombination of chromosome 1M^b^, suggesting a relaxed control of the meiotic cycle, (ii) essential recombination events may have occurred repeatedly across the individuals in a limited number of small regions, or (iii) even in the same region of chromosome 1M^b^ across all F_2_ genotypes. In all three cases, it would prevent marker ordering and construction of an LG for 1M^b^. The mapping population exhibited fitness issues, particularly in seed production, with a QTL for fertility mapped on chromosome 3M^b^ (Figure 5, Table 3). The fitness issues and growing conditions hindered reliable mapping of other traits. In the present study, we used an F_2_ biparental mapping population, where each F_2_ line was represented by a single plant. As a result, it was possible to map only the fertility trait. However, the development of an F_6_ RIL population from the F_2_ lines, using the single‐seed descent method, has been initiated. This population will enable more reliable QTL mapping of other agronomic traits that differ between the parental accessions such as leaf rust resistance, root architecture, and grain edible fiber content, in the future.

Such population‐specific chromosomal modifications and the resulting partial recombination barrier may contribute to adaptation to the local environment, potentially leading to the formation of new species (Badaeva et al., 2004; Raskina et al., 2008) and may also indicate ongoing speciation within *Ae. biuncialis*. The species already exhibits a wide range of morphological variation, on the basis of which it is sometimes divided into several subspecies (discussed in Feldman & Levy, 2023). For example, Hammer (1980) lists five subspecies: *Ae. biuncialis* var. *archipelagica* Eig, var. *macrochaeta* (Shuttl. et Huet) Eig, var. *typica* Eig, var. *velutina* Zhuk. and var. *vulgaris* Zhuk. Frequent intragenomic chromosome rearrangements have also been reported for *Ae. biuncialis* (Badaeva et al., 2004; Molnár et al., 2011). Moreover, the parental accessions MvGB382 and MvGB642 belong to different subpopulations based on a study of the genetic diversity of 86 *Ae. biuncialis* accessions originating from very different eco‐geographical habitats (Ivanizs et al., 2019). Nevertheless, further research is required to confirm any of these hypotheses. The LG for the missing 1M^b^ chromosome was artificially constructed from unmapped markers homologous to chromosome 1M and it should be used with caution.

Similarly, the LG for the short arm of chromosome 7U^b^ was not obtained (Data S1). However, in this case, cytogenetic analysis did not reveal any significant structural differences between the parental 7U^b^ chromosomes (Data S9). Further work is needed to identify and characterize the control mechanisms and genes involved in the meiotic cycle control in this cross.

### 4.1 Karyotype rearrangements

Preserved collinearity, at least to some extent, is required for the successful transfer of *Ae. biuncialis* chromatin segments to other wheat species. Compared to the CS reference genome, chromosomes 1U^b^, 2M^b^, 3M^b^, 3U^b^, 4M^b^, 5M^b^, 5U^b^, 6M^b^, and 7M^b^ show well‐preserved collinearity, whereas 2U^b^, 4U^b^, 6U^b^, and 7U^b^ are significantly rearranged.

In a previous work, Nasuda et al. (1998) detected an intra‐chromosomal translocation of a distal part of the short arm of chromosome 2M to the long arm compared to CS. Said et al. (2021) confirmed this finding and also detected a similar translocation for chromosome 2U. In the present study, such translocation is supported by markers only for chromosome 2U^b^. This is because the distal end of the long arm of chromosome 2M^b^ has only a few markers that are collinear with chromosome 2DL (Data S1). The reason for this discrepancy could be either the lack of markers in the distal part of the long arm of chromosome 2M^b^, or the presence of the translocation in the M genome of *Ae. comosa*, but not in *Ae. biuncialis*. Additionally, the distal end of the long arm of chromosome 2M^b^ is syntenic with a segment of 6U^b^‐2, suggesting a translocation of the distal region of chromosome 2M^b^L to 6U^b^‐2. This also could contribute to poor marker collinearity of chromosome 2M^b^L to 2UL in *Ae. biuncialis*.

Chromosome 4M^b^ has well‐preserved synteny with wheat chromosomal group 4, except for the distal end of the long arm of chromosome 4A, which carries the 5AL and 7BS translocations (Abrouk et al., 2017; Dvorak et al., 2018; Hernandez et al., 2011). This confirms that the ancestor of the U and M genomes diverged from the A genome before the 5AL/4AL translocation occurred.

In agreement with the findings based on single‐gene FISH mapping of orthologous genes (Said et al., 2021) and on the recently published reference genome sequence of *Ae. umbellulata* (Abrouk et al., 2023), chromosomes 4U^b^ and 6U^b^ were found to be the most rearranged. The short arm of chromosome 4U^b^ contains a translocation syntenic with the long arm of wheat chromosomal group 6. A small translocation collinear with chromosome 5D was also detected at the distal region of the long arm of 4U^b^. However, in contrast to Said et al. (2021), a translocation from the short arm of chromosome 7D to the distal end of the short arm of 4U^b^ was not detected. This was probably due to the absence of markers in this distal region.

H. Zhang et al. (1998) described synteny between the short arm of chromosome 6U and the long arm of chromosome 4D, which was confirmed by Said et al. (2021). In agreement with Said et al. (2021), we found the distal part of the short arm of chromosome 6U^b^ to be syntenic with chromosomes 4AL (inverted), 4BS, and 4DS. We also found synteny between the proximal part of the short arm of chromosome 6U^b^ and the short arms of chromosomal group 6 (inverted), which was also reported by H. Zhang et al. (1998). Similar to the findings of H. Zhang et al. (1998) and Said et al. (2021), synteny between the distal part of the long arm of chromosome 6U^b^ and the long arms of chromosomal groups 1, 2, and 7 was observed. This is consistent with the results of both H. Zhang et al. (1998) and Said et al. (2021); however, they also reported synteny between chromosome 6UL and chromosomes 2DS, 4DS, and 7DS, which we did not detect. In contrast to Danilova et al. (2014) and Said et al. (2021), who reported a paracentric inversion on the long arm of chromosome 6M, no such inversion was observed in this study.

The constructed 7U^b^ LG corresponds to a part of the long arm of chromosome 7U that was found to be syntenic with chromosomes 7DS and 3DS (Said et al., 2021; H. Zhang et al., 1998). Therefore, this LG is syntenic with the distal parts of the short arms of chromosomal group 7 and the distal parts of the short arms of chromosomal group 3. A part of the 7U^b^ LG (10–60 cM) is also syntenic with the long arm of chromosome 4A in the region of the 7BS translocation to chromosome 4AL (Hernandez et al., 2011). All of this contributes to the overall high level of rearrangement that has been observed in the U^b^ genome.

We also assessed the synteny between the chromosomes of *Ae. biuncialis*, *Ae. umbellulata* (Abrouk et al., 2023), and *Ae. comosa* (Li et al., 2024). While the U^b^ sub‐genome has well‐preserved collinearity with *Ae. umbellulata*, the M^b^ sub‐genome is rearranged (Figure 3, Data S1, S4, and S7). Similarly, the M^b^ sub‐genome is collinear with *Ae. comosa*, while the U^b^ sub‐genome is rearranged (Figure 4, Data S1, S4, and S8). This suggests that the U^b^ and M^b^ subgenomes of *Ae. biuncialis* are collinear with the genomes of diploid progenitors and the rearrangements observed in the U genome, relative to both the M genome and the D genome of *Ae. tauschii*, occurred after the divergence of *Ae. umbellulata* and *Ae. comosa*.

### 4.2 QTL mapping

Here we report the first QTL for fertility identified in *Ae. biuncialis*. *QFert.ieb‐3M^b^* was mapped to a 3.4 cM region on the long arm of chromosome 3M^b^ near the centromere (Figure 5, Table 3) with an LOD score of 11.8. The locus is collinear with a region of about 196 Mb on chromosome 3D of CS (Zhu et al., 2021) and a region of about 238 Mb on chromosome 3U of *Ae. umbellulata* TA1851 (Abrouk et al., 2023). These collinear regions are physically large because recombination is limited in the pericentromeric regions and therefore small regions in terms of cM can correspond to hundreds of Mb. The location close to the centromere may make it difficult to identify the gene(s) responsible for the trait, but it should be possible to transfer the whole region for research and breeding purposes. No QTL for fertility has been previously reported at this position. However, the QTL mapping was limited by the single phenotyping experiment since the mapping population could not be extended to the RIL stage because plants with low or zero fertility would be lost in the process, which would heavily bias the population towards the fertile plants. Therefore, it is important to interpret the results with caution. Validation of the *QFert.ieb‐3M^b^* will require further work.

## 5 CONCLUSIONS

In this study, we constructed the first genetic linkage map of *Ae. biuncialis*, a promising source of new genes and alleles for the enrichment of the bread wheat gene pool. The map contains 5663 markers, including 920 skeleton markers with an average marker density of 2.79 cM. Surprisingly, the 1M^b^ chromosomes did not recombine and the fertility of the F_2_ plants was significantly affected. This may indicate ongoing speciation within the *Aegilops* species. The U^b^ sub‐genome was found to be more rearranged compared to bread wheat, with only three chromosomes (1U^b^, 3U^b^, and 5U^b^) showing well‐preserved synteny compared to six syntenic M^b^ chromosomes. The syntenic relationships identified between the genomes of *Ae. biuncialis* and bread wheat provide a structural genomic basis for mapping and introgression of *Ae. biuncialis* genes into wheat.

## AUTHOR CONTRIBUTIONS

**Adam Lampar**: Formal analysis; visualization; writing—original draft; writing—review and editing. **András Farkas**: Investigation; writing—original draft; writing—review and editing. **László Ivanizs**: Investigation; writing—original draft; writing—review and editing. **Kitti Szőke‐Pázsi**: Investigation; writing—original draft; writing—review and editing. **Eszter Gaál**: Investigation; writing—original draft; writing—review and editing. **Mahmoud Said**: Investigation; visualization; writing—original draft; writing—review and editing. **Jan Bartoš**: Conceptualization; funding acquisition; methodology; project administration; resources; supervision; writing—original draft; writing—review and editing. **Jaroslav Doležel**: Conceptualization; data curation; funding acquisition; methodology; project administration; resources; supervision; writing—original draft; writing—review and editing. **Abraham Korol**: Formal analysis; writing—original draft; writing—review and editing. **Miroslav Valárik**: Conceptualization; data curation; funding acquisition; project administration; resources; supervision; writing—original draft; writing—review and editing. **István Molnár**: Conceptualization; data curation; funding acquisition; methodology; project administration; resources; supervision; writing—original draft; writing—review and editing.

## CONFLICT OF INTEREST STATEMENT

The authors declare no conflicts of interest.

## Supporting information




**Supplementary Data 1**: The ‘skeleton’ linkage map containing ‘skeleton’ markers, that represent groups of 2 or more co‐segregating markers, and the ‘global’ linkage map, which contains all markers.

**Supplementary Data 2**: A plot of linkage groups with only ‘skeleton’ markers representing groups of 2 or more co‐segregating markers.

**Supplementary Data 3**: Circos plots showing the syntenic relationships between the chromosomes of *Ae. biuncialis*, *T. aestivum* cv. Chinese Spring and *Ae. tauschii*.

**Supplementary Data 4**: Explanation of how detailed relationships between individual *Ae. biuncialis*, *T. aestivum* cv Chinese Spring, *Ae. tauschii*, *Ae. umbellulata* and *Ae. comosa* chromosomes can be displayed using Supplementary Data 5, 6, 7, and 8 in the Strudel software.

**Supplementary Data 5**: Data file that can be opened in the Strudel software to display syntenic relationships between the individual chromosomes of *Ae. biuncialis* and *T. aestivum* cv. Chinese Spring IWGSC 2.1 (explained in Supplementary Data 4).

**Supplementary Data 6**: Data file that can be opened in the Strudel software to display syntenic relationships between the individual chromosomes of *Ae. biuncialis* and *Ae. tauschii* ssp. *strangulata* AL8/78 Aet v6.0 (explained in Supplementary Data 4).

**Supplementary Data 7**: Data file that can be opened in the Strudel software to display syntenic relationships between the individual chromosomes of *Ae. biuncialis* and *Ae. umbellulata* TA1851 (explained in Supplementary Data 4).

**Supplementary Data 8**: Data file that can be opened in the Strudel software to display syntenic relationships between the individual chromosomes of *Ae. biuncialis* and *Ae. comosa* PI551049 (explained in Supplementary Data 4).

**Supplementary Data 9**: Cytogenetic analysis of the parental 7U^b^ chromosomes.

## Data Availability

The datasets generated during the current study are available as Supporting Information and from the corresponding author on reasonable request.
