# Supplementary material for: A linkage map of Aegilops biuncialis reveals significant genomic rearrangements compared to bread wheat
Source: Plant Genome. 2025 Feb 26;18(1):e70009. doi: 10.1002/tpg2.70009 (PMC11863542; doi:10.1002/tpg2.70009)
Supplement: Supplementary file 2 [file TPG2-18-e70009-s002.docx]

**A linkage map of *Aegilops biuncialis* reveals significant genomic rearrangements compared to bread wheat**

Adam Lampar^1,2^, András Farkas^3^, László Ivanizs^3^, Kitti Szőke-Pázsi^3^, Eszter Gaál^3^, Mahmoud Said^1,4^, Jan Bartoš^1^, Jaroslav Doležel^1^, Abraham Korol^5^, Miroslav Valárik^1#^ and István Molnár^1,3^

^1^Institute of Experimental Botany of the Czech Academy of Sciences, Centre of Plant Structural and Functional Genomics, Olomouc, Czech Republic

^2^Department of Cell Biology and Genetics, Faculty of Science, Palacký University, Olomouc, Czech Republic

^3^Department of Biological Resources, Agricultural Institute, HUN-REN Centre for Agricultural Research, Martonvásár, Hungary

^4^Field Crops Research Institute, Agricultural Research Centre, Giza, Egypt

^5^Institute of Evolution, University of Haifa, Haifa, Israel

#Correspondence: Miroslav Valárik: valarik@ueb.cas.cz

**Supplementary Data 9: Cytogenetic analysis of the parental 7U^b^ chromosomes**

FISH on mitotic metaphase 7U^b^ chromosomes from MvGB382 and MvGB642 accessions using probes for *Afa* family (green) and pSc119.2 repeats (red). Chromosomes were counterstained with DAPI (blue).
