# Supplementary material for: A linkage map of Aegilops biuncialis reveals significant genomic rearrangements compared to bread wheat
Source: Plant Genome. 2025 Feb 26;18(1):e70009. doi: 10.1002/tpg2.70009 (PMC11863542; doi:10.1002/tpg2.70009)
Supplement: Supplementary file 3 [file TPG2-18-e70009-s004.docx]

**A linkage map of *Aegilops biuncialis* reveals significant genomic rearrangements compared to bread wheat**

Adam Lampar^1,2^, András Farkas^3^, László Ivanizs^3^, Kitti Pázsi^3^, Eszter Szőke-Gaál^3^, Mahmoud Said^1,4^, Jan Bartoš^1^, Jaroslav Doležel^1^, Abraham Korol^5^, Miroslav Valárik^1#^ and István Molnár^1,3^

^1^Institute of Experimental Botany of the Czech Academy of Sciences, Centre of Plant Structural and Functional Genomics, Olomouc, Czech Republic

^2^Department of Cell Biology and Genetics, Faculty of Science, Palacký University, Olomouc, Czech Republic

^3^Department of Biological Resources, Agricultural Institute, HUN-REN Centre for Agricultural Research, Martonvásár, Hungary

^4^Field Crops Research Institute, Agricultural Research Centre, Giza, Egypt

^5^Institute of Evolution, University of Haifa, Haifa, Israel

#Correspondence: Miroslav Valárik: valarik@ueb.cas.cz

**Supplementary Data 2: Skeleton linkage map of *Ae. biuncialis***

The plotted linkage groups contain only segregating codominant SNP (skeleton) markers representing groups of 2 or more co-segregating markers. All markers are provided in Supplementary Data 1. Distances are in cM (Kosambi). Chromosome 1M^b^ is absent due to a presumed lack of recombination between the parental 1M^b^ chromosomes. Chromosomes 5U^b^ and 6U^b^ are each represented by two linkage groups. The position of the QTL for fertility (*QFert.ieb-3M^b^*) is given with a 95% confidence interval.
