# Supplementary material for: A linkage map of Aegilops biuncialis reveals significant genomic rearrangements compared to bread wheat
Source: Plant Genome. 2025 Feb 26;18(1):e70009. doi: 10.1002/tpg2.70009 (PMC11863542; doi:10.1002/tpg2.70009)
Supplement: Supplementary file 4 [file TPG2-18-e70009-s008.docx]

**A linkage map of *Aegilops biuncialis* reveals significant genomic rearrangements compared to bread wheat**

Adam Lampar^1,2^, András Farkas^3^, László Ivanizs^3^, Kitti Szőke-Pázsi^3^, Eszter Gaál^3^, Mahmoud Said^1,4^, Jan Bartoš^1^, Jaroslav Doležel^1^, Abraham Korol^5^, Miroslav Valárik^1#^ and István Molnár^1,3^

^1^Institute of Experimental Botany of the Czech Academy of Sciences, Centre of Plant Structural and Functional Genomics, Olomouc, Czech Republic

^2^Department of Cell Biology and Genetics, Faculty of Science, Palacký University, Olomouc, Czech Republic

^3^Department of Biological Resources, Agricultural Institute, HUN-REN Centre for Agricultural Research, Martonvásár, Hungary

^4^Field Crops Research Institute, Agricultural Research Centre, Giza, Egypt

^5^Institute of Evolution, University of Haifa, Haifa, Israel

#Correspondence: Miroslav Valárik: valarik@ueb.cas.cz

**Supplementary Data 4: Detailed relationships between individual *Ae. biuncialis*, *T. aestivum* cv Chinese Spring, *Ae. tauschii*, *Ae. umbellulata*, and *Ae. comosa* chromosomes**

This supplementary file shows how relationships between individual *Ae. biuncialis*, CS, *Ae. tauschii*, *Ae. umbellulata* and *Ae. comosa* chromosomes can be viewed in the Strudel software package (available at <https://ics.hutton.ac.uk/strudel/download-strudel/>) by opening Supplementary Data 5 (*Ae. biuncialis* vs *T. aestivum* cv. Chinese Spring), Supplementary Data 6 (*Ae. biuncialis* vs *Ae. tauschii* ssp. *strangulata* AL8/78 Aet v6.0), Supplementary Data 7 (*Ae. biuncialis* vs *Ae. umbellulata* TA1851) and Supplementary Data 8 (*Ae. biuncialis* vs *Ae. comosa* PI551049). Circular graphs (Figure 2, Figure 3 and Figure 4 in the main text, Supplementary Data 3) present the same data in a summarized manner.

Supplementary Data 5, 6, 7 and 8 were prepared based on BLASTN against the reference sequence of bread wheat (IWGSC 2.1), *Ae. tauschii* ssp. *strangulata* AL8/78 Aet v6.0, *Ae. umbellulata* TA1851 and *Ae. comosa* PI551049. All marker information is available in Supplementary Data 1.

Chromosome 4U^b^ of *Ae. biuncialis* is significantly rearranged compared to bread wheat. **Examples 1-3** show how this chromosome can be viewed and compared to all chromosomes of bread wheat.

**Example 1** Markers of the chromosome 4U^b^ linkage group are shown with their BLASTN obtained positions on all chromosomes of bread wheat.

**Example 2** The chromosomes can be zoomed in for a better distinction of the markers.

**Example 3** Additionally, only several chromosomes can be compared.
