# Supplementary material for: A linkage map of Aegilops biuncialis reveals significant genomic rearrangements compared to bread wheat
Source: Plant Genome. 2025 Feb 26;18(1):e70009. doi: 10.1002/tpg2.70009 (PMC11863542; doi:10.1002/tpg2.70009)
Supplement: Supplementary file 3 — Supplementary Data 3: Circos plots showing the syntenic relationships between the chromosomes of Ae. biuncialis, T. aestivum cv. Chinese Spring and Ae. tauschii. [file TPG2-18-e70009-s001.docx]

**A linkage map of *Aegilops biuncialis* reveals significant genomic rearrangements compared to bread wheat**

Adam Lampar^1,2^, András Farkas^3^, László Ivanizs^3^, Kitti Szőke-Pázsi^3^, Eszter Gaál^3^, Mahmoud Said^1,4^, Jan Bartoš^1^, Jaroslav Doležel^1^, Abraham Korol^5^, Miroslav Valárik^1#^ and István Molnár^1,3^

^1^Institute of Experimental Botany of the Czech Academy of Sciences, Centre of Plant Structural and Functional Genomics, Olomouc, Czech Republic

^2^Department of Cell Biology and Genetics, Faculty of Science, Palacký University, Olomouc, Czech Republic

^3^Department of Biological Resources, Agricultural Institute, HUN-REN Centre for Agricultural Research, Martonvásár, Hungary

^4^Field Crops Research Institute, Agricultural Research Centre, Giza, Egypt

^5^Institute of Evolution, University of Haifa, Haifa, Israel

#Correspondence: Miroslav Valárik: valarik@ueb.cas.cz

**Supplementary Data 3: Syntenic relationships between *Ae. biuncialis*, CS and *Ae. tauschii***

This supplementary file contains Circos plots displaying the relationships between the individual *Ae. biuncialis* chromosomes, CS chromosomes (IWGSC 2.1) and *Ae. tauschii* chromosomes (Aet v6.0 GCA_002575655.3), including the CS A and B sub-genome chromosomes which are not shown in the main text.

Dotted ribbons (the shorter ribbons) connect collinear segments between the same chromosomal groups. Solid ribbons (the longer ribbons) connect collinear segments between different chromosomal groups. Ribbons with horizontal lines (the shorter ribbons) or vertical lines (the longer ribbons) connect *inverted* segments between the same or different chromosomal groups, respectively. Chromosome 1M^b^ is not shown because the 1M^b^ LG could not be constructed. The CS and *Ae. tauschii* chromosomes are in Mb. For the U^b^ and M^b^ chromosomes, we arbitrarily multiplied the distances in cM by 3,000,000 to keep the figure proportions consistent; as a result, 300 Mb corresponds to 100 cM.


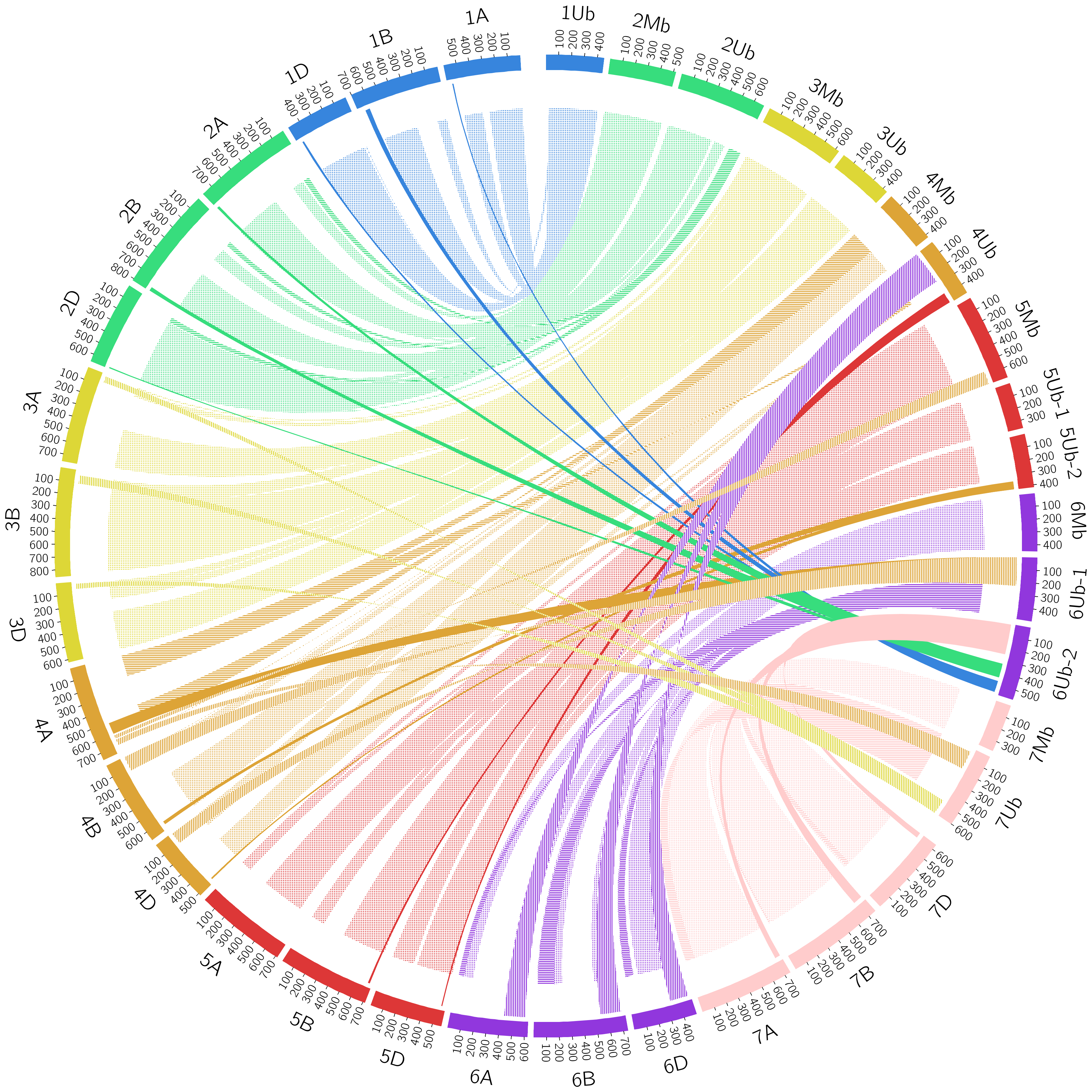


**Syntenic relationships between *Ae. biuncialis* and all CS chromosomes.**


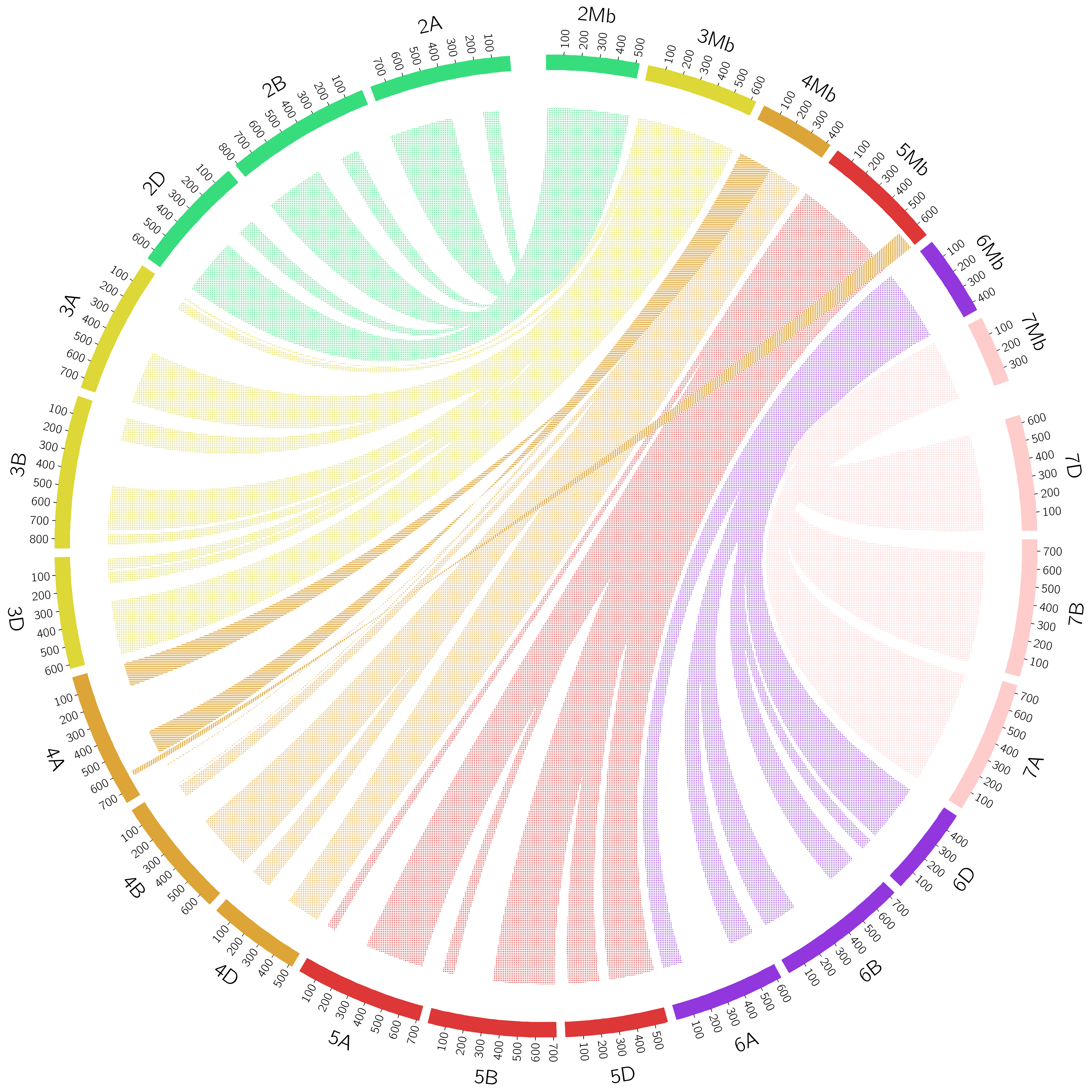


**Syntenic relationships between *Ae. biuncialis* M^b^ sub-genome and all CS chromosomes.**


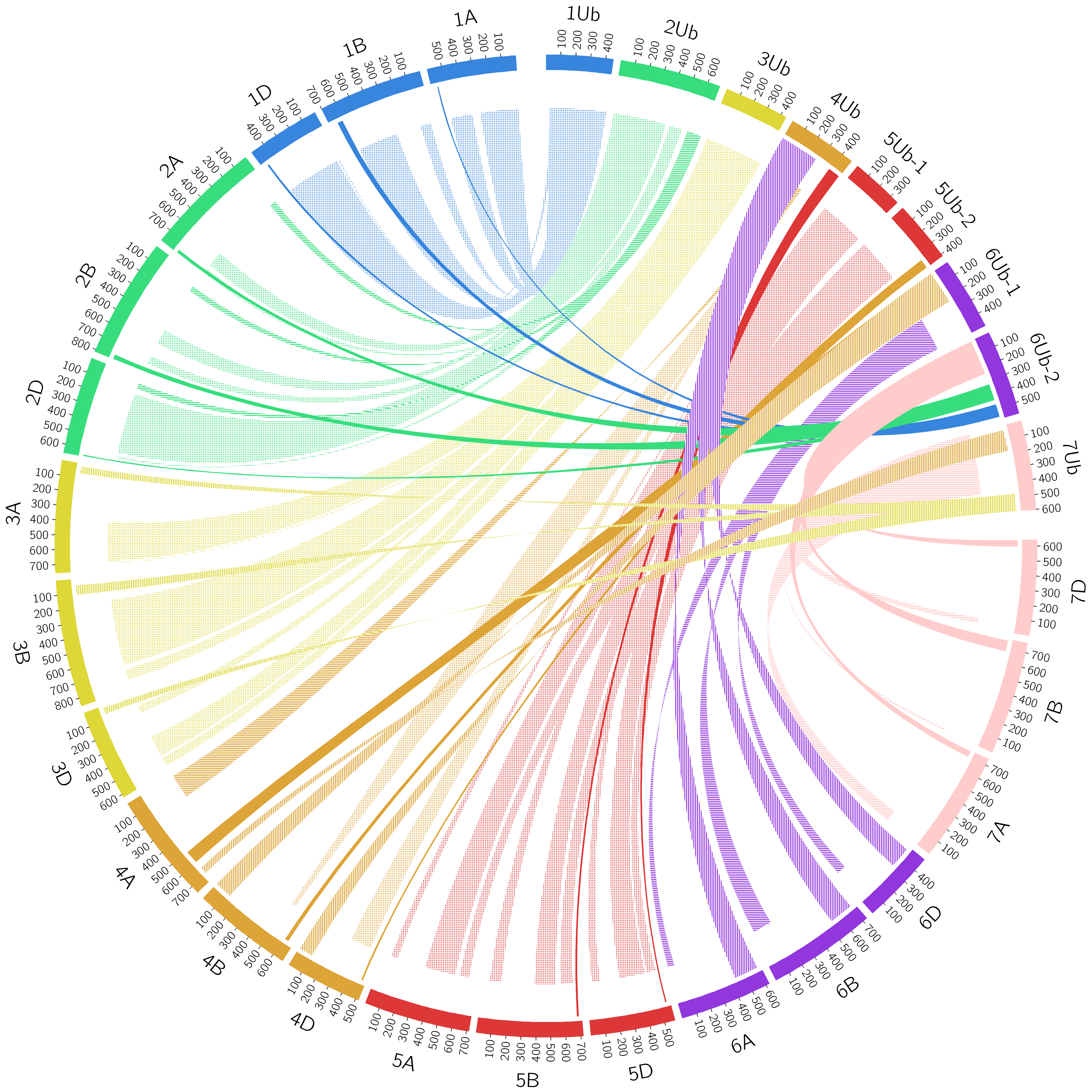


**Syntenic relationships between *Ae. biuncialis* U^b^ sub-genome and all CS chromosomes.**


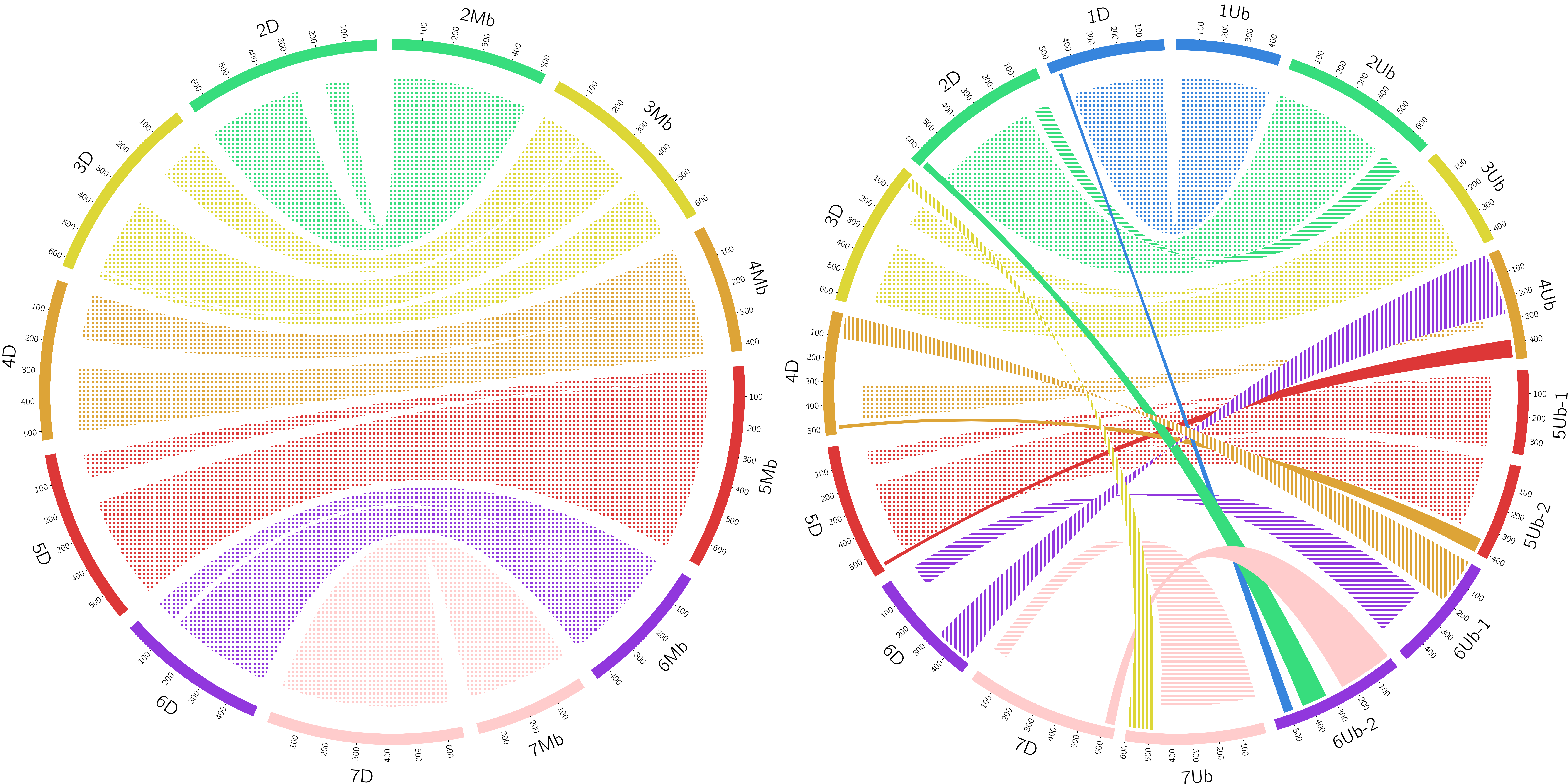


**Syntenic relationships between *Ae. biuncialis* M^b^ and U^b^ sub-genomes and *Ae. tauschii* chromosomes.**
