# Supplementary material for: A linkage map of Aegilops biuncialis reveals significant genomic rearrangements compared to bread wheat
Source: Plant Genome. 2025 Feb 26;18(1):e70009. doi: 10.1002/tpg2.70009 (PMC11863542; doi:10.1002/tpg2.70009)

**A linkage map of *Aegilops biuncialis* reveals significant genomic rearrangements compared to bread wheat**

Adam Lampar^1,2^, András Farkas^3^, László Ivanizs^3^, Kitti Szőke-Pázsi^3^, Eszter Gaál^3^, Mahmoud Said^1,4^, Jan Bartoš^1^, Jaroslav Doležel^1^, Abraham Korol^5^, Miroslav Valárik^1#^ and István Molnár^1,3^

^1^Institute of Experimental Botany of the Czech Academy of Sciences, Centre of Plant Structural and Functional Genomics, Olomouc, Czech Republic

^2^Department of Cell Biology and Genetics, Faculty of Science, Palacký University, Olomouc, Czech Republic

^3^Department of Biological Resources, Agricultural Institute, HUN-REN Centre for Agricultural Research, Martonvásár, Hungary

^4^Field Crops Research Institute, Agricultural Research Centre, Giza, Egypt

^5^Institute of Evolution, University of Haifa, Haifa, Israel

#Correspondence: Miroslav Valárik: valarik@ueb.cas.cz

**Supplementary Data 4: Detailed relationships between individual *Ae. biuncialis*, *T. aestivum* cv Chinese Spring, *Ae. tauschii*, *Ae. umbellulata*, and *Ae. comosa* chromosomes**

This supplementary file shows how relationships between individual *Ae. biuncialis*, CS, *Ae. tauschii*, *Ae. umbellulata* and *Ae. comosa* chromosomes can be viewed in the Strudel software package (available at <https://ics.hutton.ac.uk/strudel/download-strudel/>) by opening Supplementary Data 5 (*Ae. biuncialis* vs *T. aestivum* cv. Chinese Spring), Supplementary Data 6 (*Ae. biuncialis* vs *Ae. tauschii* ssp. *strangulata* AL8/78 Aet v6.0), Supplementary Data 7 (*Ae. biuncialis* vs *Ae. umbellulata* TA1851) and Supplementary Data 8 (*Ae. biuncialis* vs *Ae. comosa* PI551049). Circular graphs (Figure 2, Figure 3 and Figure 4 in the main text, Supplementary Data 3) present the same data in a summarized manner.

Supplementary Data 5, 6, 7 and 8 were prepared based on BLASTN against the reference sequence of bread wheat (IWGSC 2.1), *Ae. tauschii* ssp. *strangulata* AL8/78 Aet v6.0, *Ae. umbellulata* TA1851 and *Ae. comosa* PI551049. All marker information is available in Supplementary Data 1.

Chromosome 4U^b^ of *Ae. biuncialis* is significantly rearranged compared to bread wheat. **Examples 1-3** show how this chromosome can be viewed and compared to all chromosomes of bread wheat.

**Example 1** Markers of the chromosome 4U^b^ linkage group are shown with their BLASTN obtained positions on all chromosomes of bread wheat.


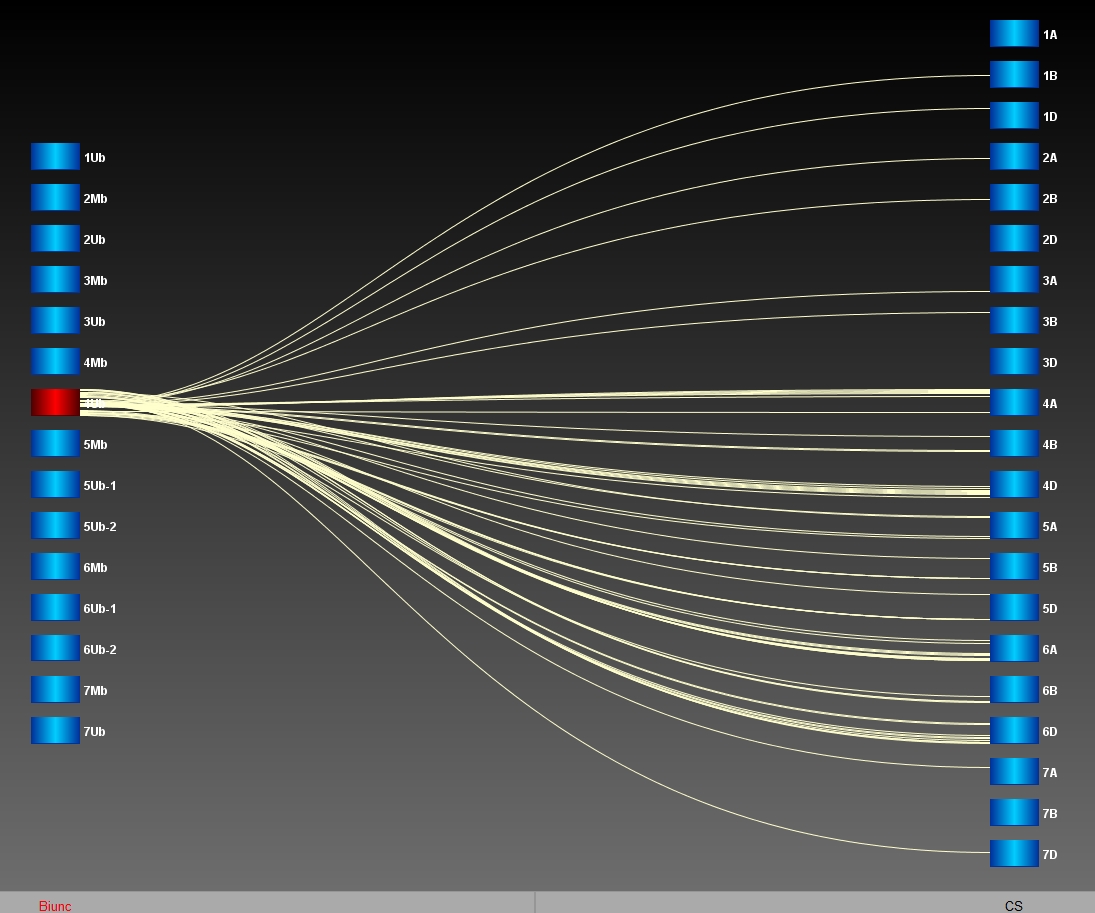


**Example 2** The chromosomes can be zoomed in for a better distinction of the markers.


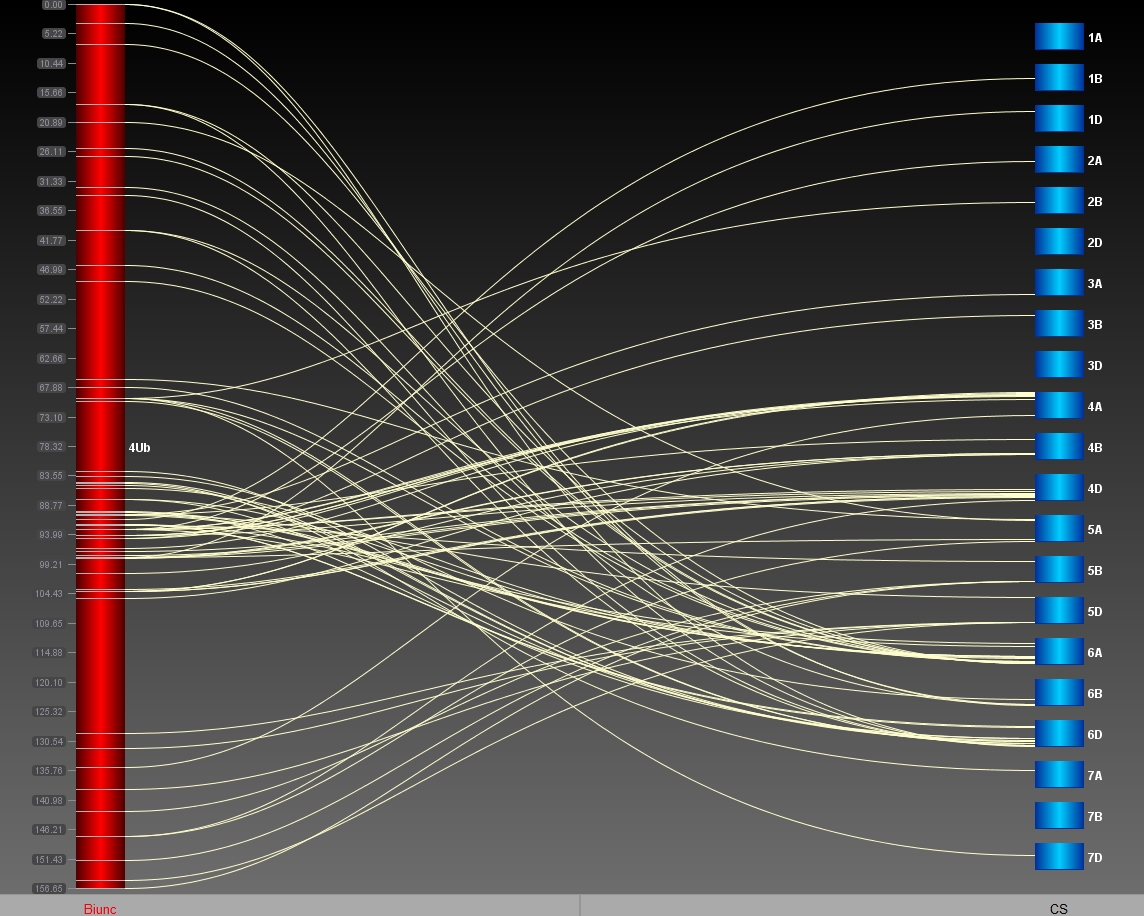


**Example 3** Additionally, only several chromosomes can be compared.


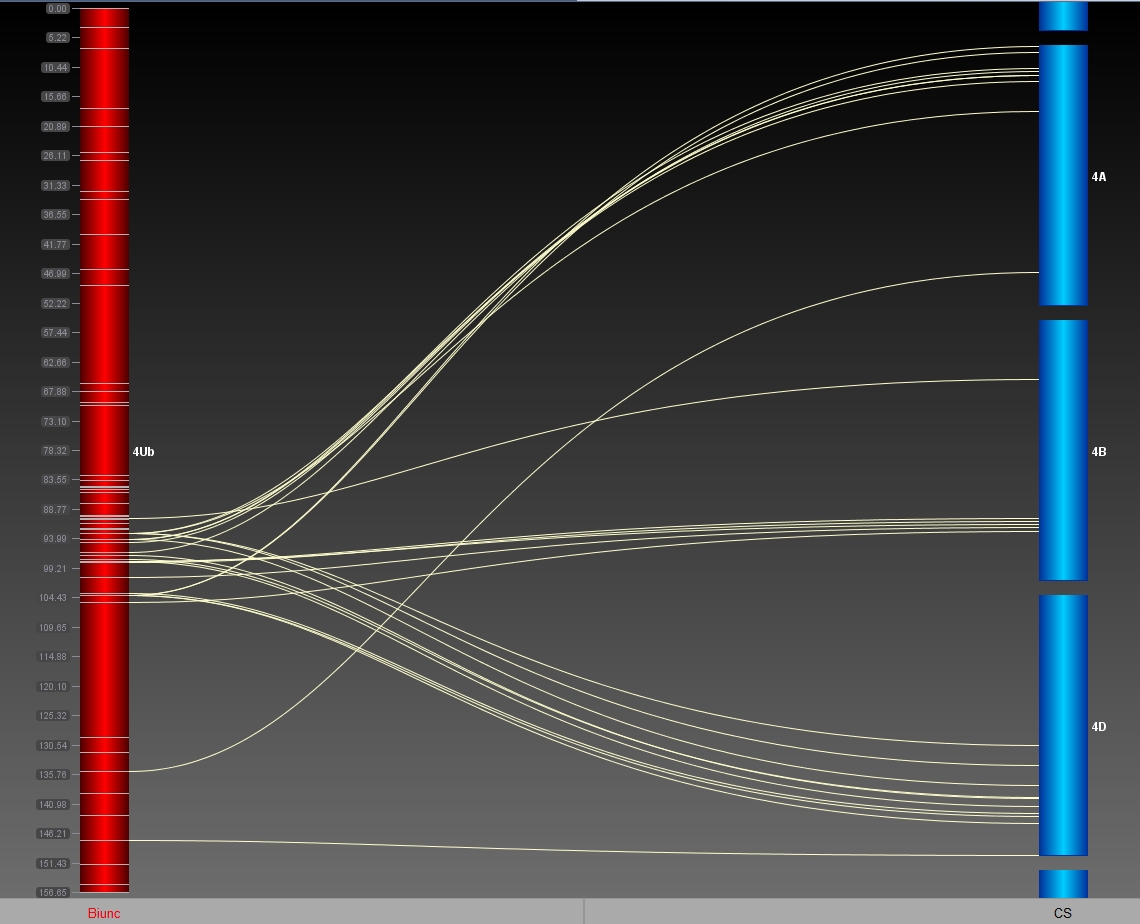

Supplement: Supplementary file 4 — Supplementary Data 4: Explanation of how detailed relationships between individual Ae. biuncialis, T. aestivum cv Chinese Spring, Ae. tauschii, Ae. umbellulata and Ae. comosa chromosomes can be displayed using Supplementary Data 5, 6, 7, and 8 in the Strudel software. [file TPG2-18-e70009-s008.docx]
